# *Arabidopsis* seed germination speed is controlled by SNL histone deacetylase-binding factor-mediated regulation of *AUX1*

**DOI:** 10.1038/ncomms13412

**Published:** 2016-11-11

**Authors:** Zhi Wang, Fengying Chen, Xiaoying Li, Hong Cao, Meng Ding, Cun Zhang, Jinghong Zuo, Chaonan Xu, Jimei Xu, Xin Deng, Yong Xiang, Wim J. J. Soppe, Yongxiu Liu

**Affiliations:** 1 Key Laboratory of Plant Molecular Physiology, Institute of Botany, Chinese Academy of Sciences, Beijing 100093, China; 2 College of Life Sciences, University of Chinese Academy of Sciences, Beijing 100049, China; 3 Key Laboratory of Plant Resources, Institute of Botany, Chinese Academy of Sciences, Beijing 100093, China; 4 Department of Plant Breeding and Genetics, Max Planck Institute for Plant Breeding Research, 50829 Cologne, Germany; 5 Institute of Molecular Physiology and Biotechnology of Plants (IMBIO), University of Bonn, 53115 Bonn, Germany

## Abstract

Histone acetylation is known to affect the speed of seed germination, but the molecular regulatory basis of this remains ambiguous. Here we report that loss of function of two histone deacetylase-binding factors, SWI-INDEPENDENT3 (SIN3)-LIKE1 (SNL1) and SNL2, results in accelerated radicle protrusion and growth during seed germination. AUXIN RESISTANT 1 (AUX1) is identified as a key factor in this process, enhancing germination speed downstream of SNL1 and SNL2. *AUX1* expression and histone H3 acetylation at lysines 9 and 18 is regulated by SNL1 and SNL2. The D-type cyclins encoding genes *CYCD1;1* and *CYCD4;1* display increased expression in *AUX1* over-expression lines and the *snl1snl2* double mutant. Accordingly, knockout of CYCD4;1 reduces seed germination speed of *AUX1* over-expression lines and *snl1snl2* suggesting the importance of cell cycling for radicle protrusion during seed germination. Together, our work identifies AUX1 as a link between histone acetylation mediated by SNL1 and SNL2, and radicle growth promoted by CYCD1;1 and CYCD4;1 during seed germination.

## Abstract (web summary)

Histone acetylation influences the speed of seed germination. Here, Wang *et al*. show that loss of the SNL1/SNL2 histone deacetylase binding factors accelerates seed germination and provide evidence that they act by regulating the expression of *AUX1* which in turn influences cell division.

## Graphical abstract

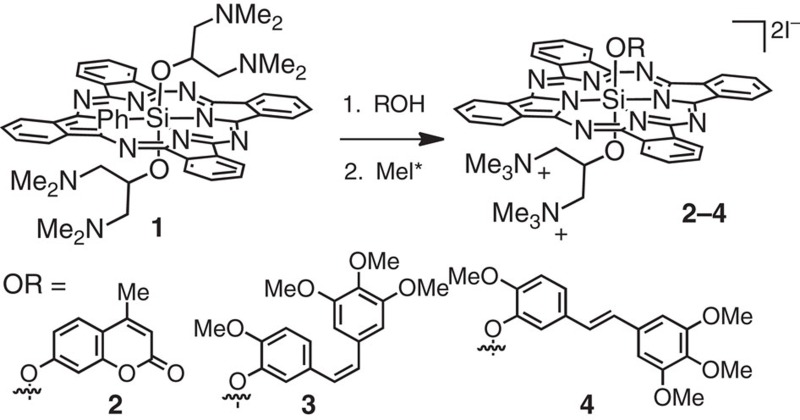

Germination is a critical step in the life cycle of seed plants converting a quiescent seed to a highly active seedling. Seed germination is required for the next generation to enter the ecosystem, and its proper timing ensures offspring propagate under suitable conditions. In agriculture, fast and uniform seed germination is also necessary for high crop yield. Seeds can germinate after the release of dormancy by extended storage (after-ripening) or imbibition at species-specific temperatures (stratification). Germination includes a subsequent series of events starting with the uptake of water by the dry seed and finishing with the elongation of the embryonic axis and the protrusion of the radicle^1^. Seed germination is a complex process regulated by genetic and environmental factors^2^,^3^,^4^,^5^.

Studies have identified crucial roles for abscisic acid (ABA) and gibberellic acid (GA) in seed germination^6^,^7^. The application of exogenous ABA inhibits seed germination and mutants defective in ABA biosynthesis or signalling have enhanced germination efficiency^6^,^7^. The ABSCISIC ACID INSENSITIVE (ABI) factors, ABI1, ABI2, ABI3, ABI4 and ABI5, act in the ABA inhibition of seed germination^8^,^9^. Conversely, GA promotes seed germination. GA-deficient mutants such as *ga1* and *ga2* show a delay or absence of seed germination^10^,^11^. GA signalling requires the DELLA proteins REPRESSOR OF GA (RGA), GIBBERELLIC ACID INSENSITIVE (GAI) and RGA-LIKE 2 (RGL2), which play negative roles in seed germination^6^,^12^,^13^.

Apart from ABA and GA additional hormones like auxin play a role in germination^5^. Auxin has been shown to function both positively and negatively in seed germination depending on its dose. Exogenous application of high auxin concentrations from 0.3 to 1 μm indole-3-acetic acid (IAA) can inhibit seed germination in *Arabidopsis*^14^,^15^. In contrast application of low concentrations of auxin from 0.03 to 3 nM IAA can promote seed germination and seedling establishment^16^,^17^. Genetic studies have also provided evidence for the involvement of auxin pathway factors in both germination completion and early seedling establishment^18^,^19^. AUXIN RESPONSE FACTORS (ARF) 10/16 act as positive regulators of the ABA signal pathway by regulating expression of *ABI3*, and the *arf10arf16* double mutant showed insensitivity for seed germination to ABA^18^. Transgenic seeds expressing a miR160-resistant form of *ARF10* (*mARF10*) were hypersensitive to germination inhibition by exogenous ABA, whereas ectopic expression of *miR160* resulted in a reduced sensitivity to ABA^15^. Interestingly, another *ARF* gene, *ARF2*, was induced by ABA, and the *arf2* mutants displayed enhanced ABA sensitivity during seed germination. Conversely, over-expression of *ARF2* decreased the inhibition of seed germination by ABA^19^, suggesting that ARF2 is involved in seed germination by repressing the ABA signalling pathway. Transcriptomic studies have shown that RNAs encoding the auxin transporters AUXIN RESISTANT 1 (AUX1), PIN-FORMED 2 (PIN2) and PIN7 were highly upregulated in response to GA treatment of *ga1* mutant seeds^20^. In addition, both efflux and influx transporters are upregulated in after-ripened seeds compared with dormant seeds^21^, suggesting that auxin transporters might be important for seed germination. AUX1 is required for ABA inhibition of seed germination, loss-of-function mutants of AUX1 showed increased ABA resistance^22^. These results indicate that distinct auxin signalling pathways are involved in seed germination by affecting ABA and/or GA signal pathways. These functions of auxin are commonly achieved through the auxin transport carriers in the root tip among which AUX1 has an important role^23^.

Apart from plant hormones, chromatin factors have been shown to control seed germination. PICKLE (PKL), a CHD3 class SWI/SNF chromatin-remodelling factor, is involved in repression of embryonic traits during germination. *PKL* transcript is absent in dry seeds and is initiated on seed imbibition^24^. The *pkl* mutants showed hypersensitivity to ABA-mediated repression of germination, indicating that PKL acts as a negative factor of ABA signalling during seed germination^25^. Mutants in FERTILIZATION-INDEPENDENT ENDOSPERM, an essential component of the polycomb repressive complex 2, display a genome-wide reduction in histone 3 lysine 27 trimethylation (H3K27me3) and exhibit increased seed germination defects^26^. Mutations in *KRYPTONITE/SU(VAR)3-9 HOMOLOG4* (*KYP/SUVH4*), encoding a histone methyltransferase that is required for histone 3 lysine 9 dimethylation (H3K9me2)^27^, result in decreased seed germination in response to ABA and the GA biosynthesis inhibitor paclobutrazol^28^. The histone arginine demethylases, JUMONJI DOMAIN-CONTAINING PROTEIN 20 (JMJ20) and JMJ22, act redundantly as positive regulators of seed germination by removing repressive histone arginine methylation at *GA3ox1/GA3ox2* to regulate GA levels^29^. *Arabidopsis* AL PHD–PRC1 complexes have been shown to promote seed germination by switching the chromatin state from H3K4me3 to H3K27me3 to repress seed developmental genes such as *ABI3*, *DELAY OF GERMINATION 1* (*DOG1*) and *CHOTTO1* (*CHO1*)^30^. Recently, a role for histone acetylation in seed germination was demonstrated by the increased seed germination speed phenotype of the histone deacetylase 9 (*hda9*) mutant^31^.

We previously showed that histone deacetylation mediated by SNL1 and SNL2 is involved in the regulation of seed dormancy by affecting the ABA/ethylene antagonism^32^. Here we report that SNL1 and SNL2 also regulate radicle promotion and early growth in a manner dependent on *AUX1*. We show that SNLs repress *AUX1* expression and histone deacetylation of H3K9K18ac. Loss of function of SNL1 and SNL2 causes significantly increased transcript levels of auxin-related genes, including *AUX1*. Genetic evidence further confirms that AUX1 is a key factor with a positive role in regulating seed germination downstream of SNL1/SNL2. Moreover, we identify potentially important roles for the D-type cyclins CYCD1;1 and CYCD4;1 in radicle protrusion and early growth mediated by AUX1 and SNL1/SNL2.

## Results

### SNL1 and SNL2 negatively regulate radicle protrusion

**Figure 1:**
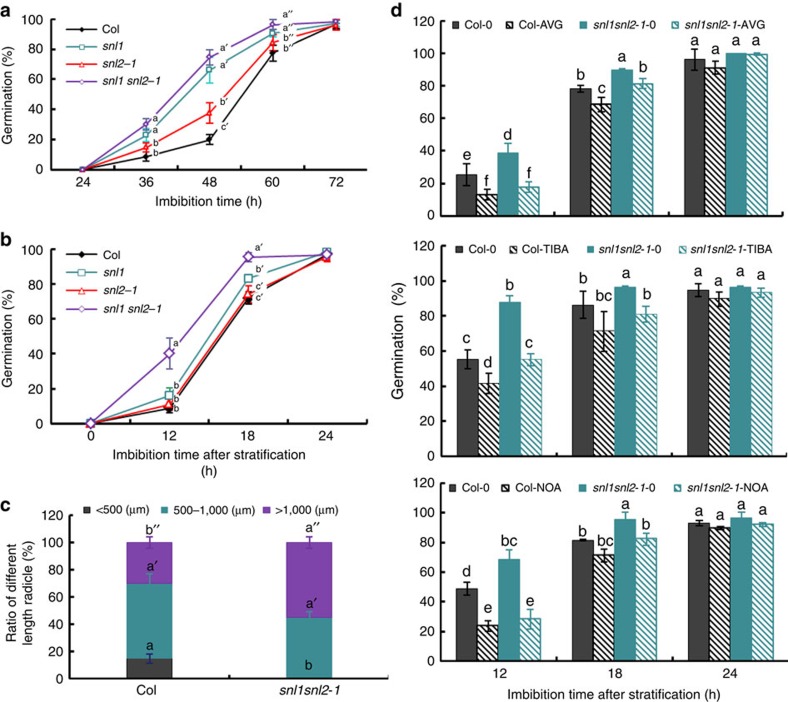
Seed germination of *snl* mutants and their response to auxin pathway inhibitors. (**a**,**b**) Germination phenotypes of wild-type Col, *snl1*, *snl2-1* and *snl1snl2*. Seeds were after-ripened for 4 months at room temperature before the germination experiment to complete dormancy release (**a**) or after-ripened and subsequently stratified for 3 days at 4 °C (**b**). Percentages of seed germination are means (±s.d.) based on the seeds from eight individual plants. Different letters at the same timepoint indicate a significant difference determined by Tukey's honestly significant difference (HSD) test (*P*<0.05). (**c**) Radicle length after 2 days imbibition of after-ripened seeds from Col and *snl1snl2-1.* The radicles were divided in three groups according to their size. Data are means (±s.e.) of two independent experiments with three technical replicates (*N*=2, *n*=3 × 30 seeds). Different letters indicate a significant difference determined by Tukey's HSD test (*P*<0.05). (**d**) Germination percentage of Col and *snl1snl2* after exogenous application of auxin synthesis inhibitor AVG (top panel, 40 μM) and auxin transport inhibitors TIBA (middle panel, 25 μM) and NOA (bottom panel, 25 μM). The seeds were after-ripened and stratified. Percentages of seed germination were shown after 12, 18 and 24 h imbibition (±s.d., *n*=8). Different letters indicate a significant difference determined by Tukey's HSD test (*P*<0.05).

We have previously shown that SNL1 and SNL2 are components of the SNL-HDA19 histone deacetylation complex and that they regulate seed dormancy by deacetylating histone H3K9/18 sites of ABA and ethylene signalling-related genes^32^. We analysed seed germination of *snl1*, *snl2* single and *snl1snl2* double mutants in more detail and observed accelerated radicle protrusion and growth of after-ripened seeds with fully released seed dormancy. Although the mutants displayed a faster radicle protrusion than the wild type, all of them germinated 100% after 3 days (Fig. 1a). This phenotype was confirmed with after-ripened seeds that had been stratified at 4 °C for 3 days (Fig. 1b and Supplementary Fig. 1). These observations indicated that SNL1 and SNL2 regulate seed germination independent of dormancy control. The enhanced germination of *snl1snl2* was analysed in more detail by measuring radicle length after 2 days imbibition. In *snl1snl2*, 60% of the radicles were longer than 1,000 μm and 40% in between 500 and 1,000 μm. These percentages were, respectively, 30 and 50% in Col (Fig. 1c), confirming the faster radicle growth in *snl1snl2*.

### Auxin-related genes show enhanced expression in *snl1snl2*

We previously performed an RNA-sequencing (RNA-seq) analysis of 12 h imbibed freshly collected seeds of the *snl1snl2* mutant and Col to identify genes with altered transcript levels^32^. A detailed analysis of these data showed that a high number of auxin-related genes had significantly increased transcript levels in the *snl1snl2* mutant compared with wild-type Col (Supplementary Table 1). The role of auxin in the germination of *snl1snl2* was analysed by imbibing seeds in the presence of the auxin synthesis inhibitor aminoethoxyvinylglycine (AVG)^33^ and the auxin transport inhibitors 2,3,5-triidobenzoid acid (TIBA) and 1-naphthoxyacetic acids (1-NOA). Interestingly, all three inhibitors caused a decrease in germination speed for both *snl1snl2* double mutant and wild-type Col. In response to AVG, TIBA and NOA, the germination percentages were reduced from 25 to 13%, from 55 to 41% and from 49 to 24% for Col, and from 39 to 17%, from 88 to 55% and from 68 to 28% for *snl1snl2*, respectively, at 12 h after imbibition (Fig. 1d). In general, this inhibition was stronger for the *snl1snl2* double mutant compared with wild type. Because a positive influence of auxin on seed germination has been reported^16^,^17^,^19^, we speculated that the accelerated seed germination of the *snl* mutants is caused by an enhanced auxin response.

### Auxin synthesis and transport are enhanced in *snl1snl2*

**Figure 2:**
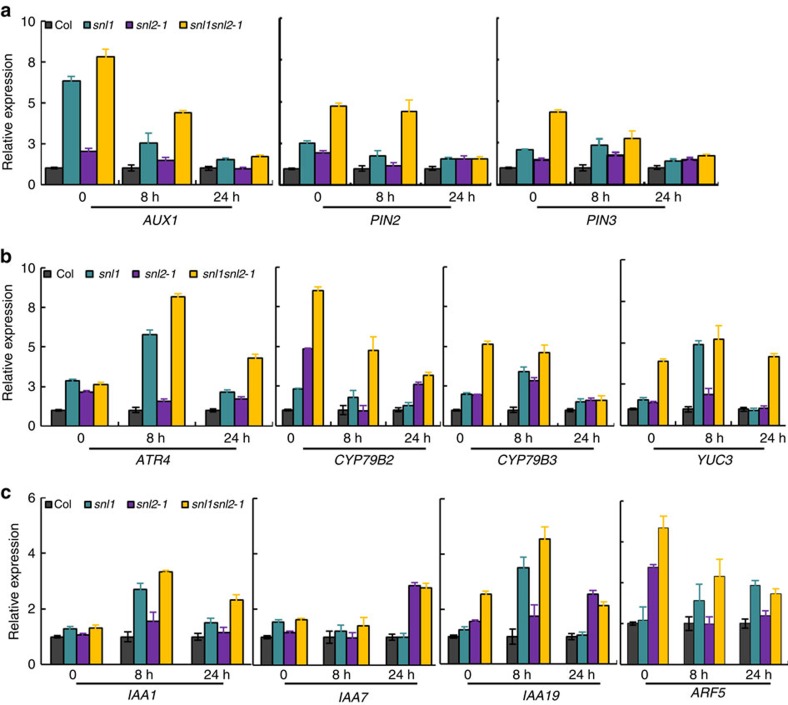
The *snl* mutants show enhanced transcript levels of auxin-related genes during seed imbibition. The relative expression of (**a**) auxin transport-related genes, (**b**) auxin synthesis-related genes and (**c**) auxin signalling-related genes in seeds of Col, *snl1*, *snl2* and *snl1snl2* measured by qRT–PCR. Each experiment had three biological repeats and the average value is shown with s.e. Transcript levels were normalized to the level of *ACTIN8*. The relative transcript level in Col is set as one. The seeds for RNA isolation were fully after-ripened and imbibed for 0, 8 and 24 h.

Quantitative reverse transcription–PCR (qRT–PCR) was used to confirm genes associated with auxin synthesis, transport and signalling had increased transcript levels in *snl* mutant seeds. Almost all tested genes showed significantly increased transcript levels in after-ripened dry and 8 h imbibed *snl1* and *snl1snl2* seeds compared with wild-type Col (Fig. 2). However, after 24 h imbibition several of these genes showed similar transcript levels in *snl* mutants and wild type. In general, we observed a positive correlation between transcription levels of auxin pathway genes and seed germination speed in the *snl1snl2* double mutant (Figs 1 and 2)^17^.

**Figure 3:**
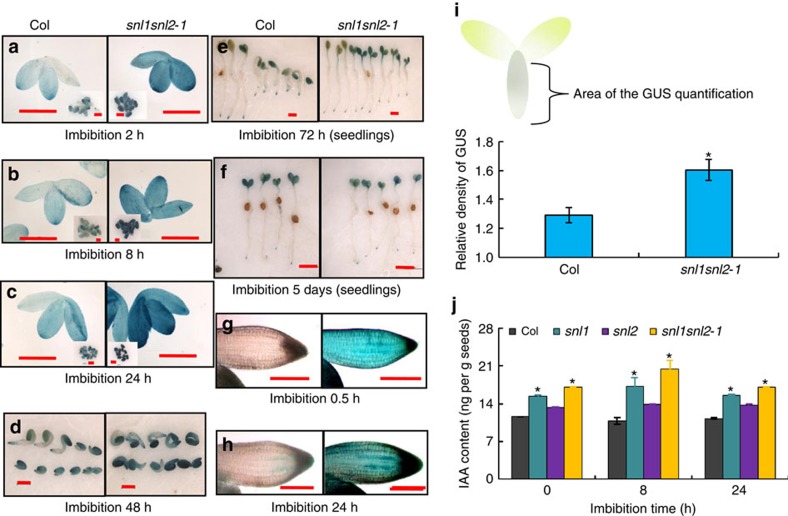
The *snl1snl2* mutant accumulates high levels of auxin. (**a**–**h**) Signals of the auxin reporter DR5::GUS were visualized by GUS assay and imaged in embryos and seedlings after 0.5 h–5-day imbibition of Col and *snl1snl2* seeds. Scale bars, 500 μm (**a**–**f**); 200 μm (**g**,**h**). (**i**) The GUS intensity in the radicle of Col and *snl1snl2* was measured by ImageJ software, the background was set as one and relative levels are shown. Error bars denote s.d. (*n*>30). The radicle area used for GUS quantification is indicated in the upper diagram. The asterisk indicates a significant difference according to Student's *t*-test (*P*<0.05). (**j**) The IAA content in dry, 8 and 24 h imbibed seeds of Col and *snl1snl2* was determined by GC–MS. Three independent experiments were performed and the average value is shown with s.d. The asterisk indicates a significant difference according to Student's *t*-test (*P*<0.05).

To further study the role of SNL1 and SNL2 in the auxin pathway during seed germination, the auxin-responsive reporter *DR5::GUS* was introduced into the *snl* mutants. The abundance of the GUS signal was analysed during seed germination and early seedling establishment. DR5::GUS signals in the embryo were significantly higher in *snl1* and *snl1snl2* compared with Col wild type and *snl2* during radicle protrusion and early growth, but not in the mature seedling (Fig. 3a–f, and Supplementary Figs 2 and 3). In particular, DR5::GUS levels were increased in the radicle tip of *snl1snl2* during seed imbibition before radicle protrusion (Fig. 3g,h). Quantification of the GUS signal by ImageJ software confirmed these observations (Fig. 3i). Moreover, quantification of IAA levels using gas chromatography–mass spectrometry (GC–MS) showed significantly higher levels in the seeds of *snl1* and *snl1snl2* compared with Col (Fig. 3j). Overall, the results indicated that SNL1 and SNL2 negatively regulate auxin levels during seed germination, which might affect germination speed.

### Auxin promotes seed germination and activates cell cycling

**Figure 4:**
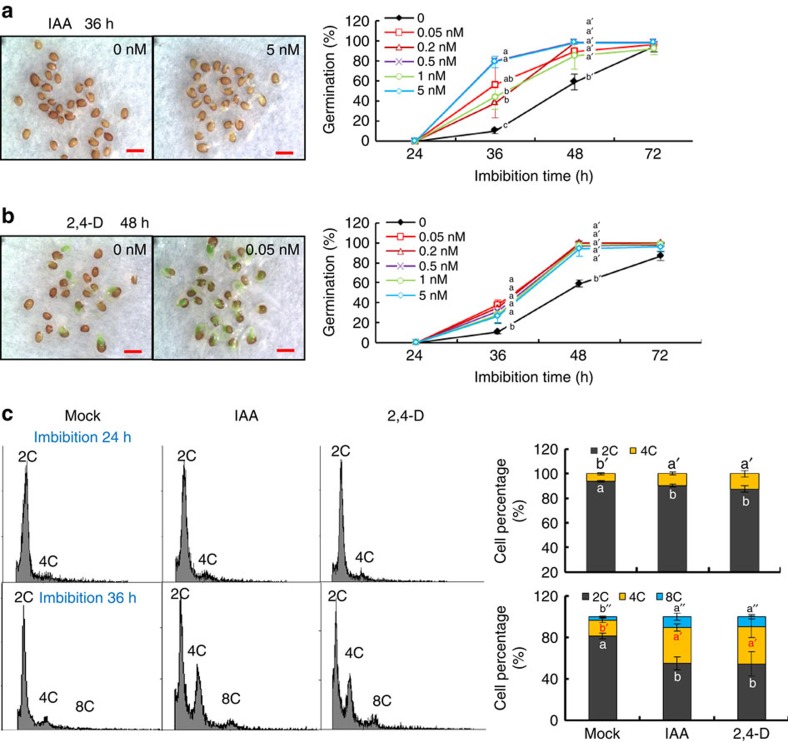
Auxin treatment with low doses stimulates seed germination and enhances DNA content. (**a**,**b**) Germinating seeds of Col (left) are shown after 36 h imbibition in 5 nM IAA or mock solution (**a**) and 48 h imbibition in 0.05 nM 2,4-D or mock solution (**b**). The graphs on the right show seed germination percentages during imbibition with different concentrations of (**a**) IAA and (**b**) 2,4-D (0.05–5 nM) treatment. Different letters at the same timepoint indicate a significant difference determined by Tukey's honestly significant difference (HSD) test (*P*<0.05). Scale bars, 1 mm. (**c**) Changes in nuclear ploidy levels during seed imbibition. Histograms representing DNA content of embryo cells during seed imbibition (left). Fully after-ripened wild-type Col seeds were imbibed for 24 h (upper panel) or 36 h (lower panel) in a mock solution, 5 nM IAA or 0.05 nM 2,4-D. 2C peak, G1 DNA content; 4C peak, G2 DNA content; 8C and 16C peaks, endoreduplicating cells. The graphs in the right show a quantification of the cell numbers at the different stages of the cell cycle based on the histograms. The bars indicate s.d. of three biological replicates and different letters indicate a significant difference determined by Tukey's HSD test (*P*<0.05).

To study the role of auxin during seed germination, fully after-ripened Col seeds were imbibed in the presence of low or high concentrations of IAA or the auxin analogue 2,4-dichlorophenoxyacetic acid (2,4-D). Both IAA and 2,4-D promoted germination speed at low doses (0.05–5 nM) in stratified and non-stratified seeds (Fig. 4a,b and Supplementary Fig. 4a), and decreased germination speed at high doses (0.05–5 μm) (Supplementary Fig. 4b,c). Imbibition of seeds in 5 nM IAA increased the DR5::GUS signal in the radicle tip (Supplementary Fig. 5), which implied that external IAA treatment enhanced the internal auxin response level.

Auxin can regulate plant development by inducing cell elongation and division^34^,^35^. Radicle elongation of *Arabidopsis* seeds does not require cell division, but cell elongation of the hypocotyl-radicle transition zone. The increase of cell size in the region of elongation is accompanied by an increase in the proportion of 4C and endopolyploid (8C and 16C) nuclei^36^. We analysed the DNA content of embryo cells by flow cytometry and detected enhanced levels of 4C and 8C nuclei in 24 and 36 h imbibed seeds in the presence of 5 nM IAA or 2,4-D (Fig. 4c). These results suggest that auxin may promote germination speed by stimulating cell mitosis or endocycle.

### AUX1 plays a key role in seed germination mediated by SNLs

**Figure 5:**
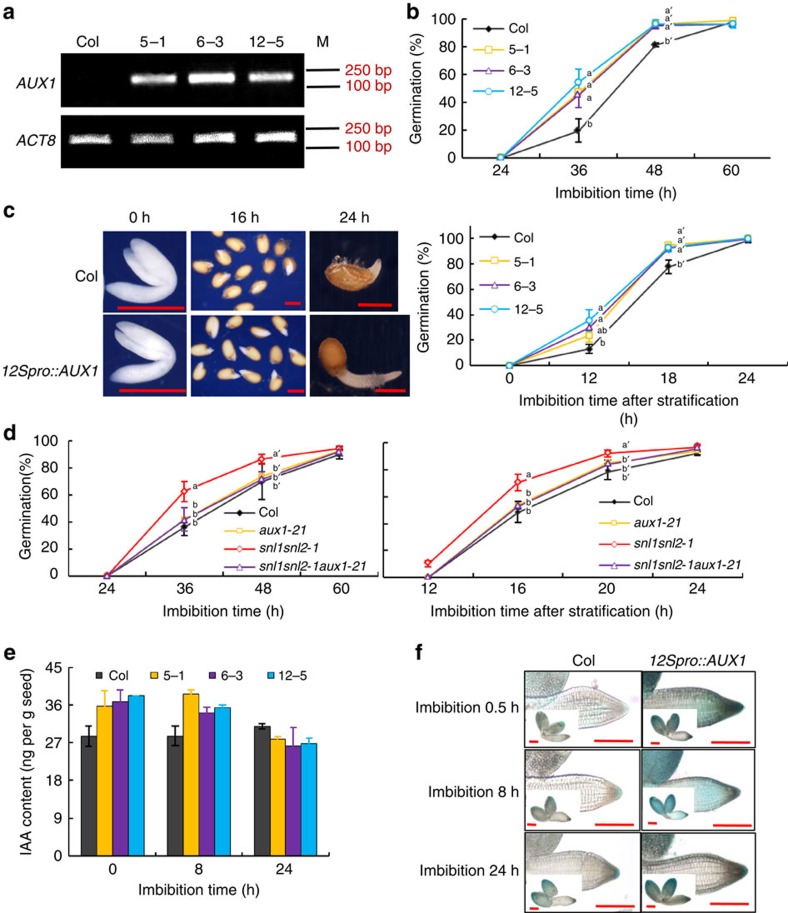
Overexpression of *AUX1* causes increased auxin level and seed germination. (**a**) *AUX1* transcript levels in dry seeds of three independent homozygous transgenic lines containing *12Spro::AUX1* (*5-1*, *6-3* and *12-5*). Semi-quantitative PCR was used to detect *AUX1* transcript levels and the *ACT8* gene was used as control. M, DNA molecular weight marker. (**b**) Seed germination during imbibition of fully after-ripened seeds from Col and *12Spro::AUX1* lines (*5-1*, *6-3* and *12-5*). Percentages of seed germination are means (±s.d.) based on eight individual plants. Different letters at the same timepoint indicate a significant difference determined by Tukey's honestly significant difference (HSD) test (*P*<0.05). (**c**) Germination phenotype of after-ripened stratified seeds from wild-type Col and three *12Spro::AUX1* homozygous transgenic lines. The left panel shows the photos of embryos and seeds from Col and the *12Spro::AUX1* homozygous line *5-1* at different imbibition times. The right panel shows germination percentages during imbibition. More than eight individual plants were counted and the average value is shown with the s.d. Different letters at the same timepoint indicate a significant difference determined by Tukey's (HSD) test (*P*<0.05). Scale bars, 500 μm. (**d**) Germination phenotypes of after-ripened seeds without (left) or with stratification (right) from wild-type, *aux1-21*, *snl1snl2-1* and the triple mutant *snl1snl2-1aux1-21*. Percentages of seed germination are means (±s.d.) based on eight individual plants. Different letters at the same timepoint indicate a significant difference determined by Tukey's HSD test (*P*<0.05). (**e**) The IAA content in dry, 8 and 24 h imbibed seeds of Col and three *12Spro::AUX1* homozygous transgenic lines was quantified by GC–MS. Three independent experiments were performed and the average value is shown with the s.d. (**f**) Photos showing GUS staining of radicles and embryos (inserts) from Col and a *12Spro::AUX1* transgenic line, both containing the auxin reporter gene *DR5::GUS*. Seeds were imbibed for 0.5, 8 and 24 h. The experiment was performed twice with 15 embryos and representative photos are shown. Scale bars, 200 μm.

Auxin is likely to be important for the increased germination speed of the *snl1snl2* double mutant (Figs 1, 2, 3). We next aimed to identify key factors in the auxin pathway involved in the regulation of germination speed downstream of SNL1 and SNL2. Mutants of auxin-related genes (for example, *PIN2*, *PIN3*, *CYP79B2*, *CYP79B3* and *YUC3*) that showed enhanced transcript levels in *snl1snl2* (Supplementary Table 1) did not show obvious seed germination phenotypes. However, the *aux1-21* and *aux1-22* mutants did show weakly decreased germination of fresh seeds compared with wild type (Supplementary Fig. 6a). This phenotype was not visible in after-ripened stratified seeds (Supplementary Fig. 6b), suggesting that AUX1 is a weak seed dormancy regulator. The *aux1-T* mutant, another mutant allele of *AUX1* (ref. 37), behaved similarly (Supplementary Fig. 6b). RNA-seq data and qRT–PCR results showed that auxin-related genes were upregulated in *snl1snl2* (Fig. 2 and Supplementary Table 1)^32^. Therefore, we cloned *AUX1*, *ARF5*, *ATR4*, *CYP79B2*, *YUC3* and so on (Supplementary Table 1) under the 12S promoter to obtain high transcript levels in seeds. Transgenic plants containing these constructs were obtained and their seed germination phenotypes were analysed. After-ripened and stratified seeds of transgenic lines with *12Spro::AUX1* displayed significantly accelerated radicle protrusion (Fig. 5a–c). Interestingly, combining the *aux1-21* mutant with the *snl1snl2* double mutant restored the enhanced germination of *snl1snl2* back to wild-type levels (Fig. 5d). This implied that AUX1 has an important role in the regulation of germination speed downstream of SNL1 and SNL2 (Fig. 5d). The transgenic lines that over-express other auxin-related genes did not show seed germination phenotypes.

Previous studies have shown that loss of AUX1 function resulted in decreased auxin levels^37^,^38^,^39^. Accordingly, we observed enhanced IAA levels in dry and imbibed seeds of *AUX1* over-expression lines using GC–MS (Fig. 5e). Furthermore, introduction of the auxin reporter *DR5::GUS* into the *12Spro::AUX1* transgenic line showed enhanced GUS signals in the *12Spro::AUX1* radicle (Fig. 5f). These results suggest that AUX1 may be involved in radicle growth and seed germination by transporting auxin and modifying its accumulation in the radicle tip.

### AUX1 accumulate in the radicle tip of *snl1snl2*

**Figure 6:**
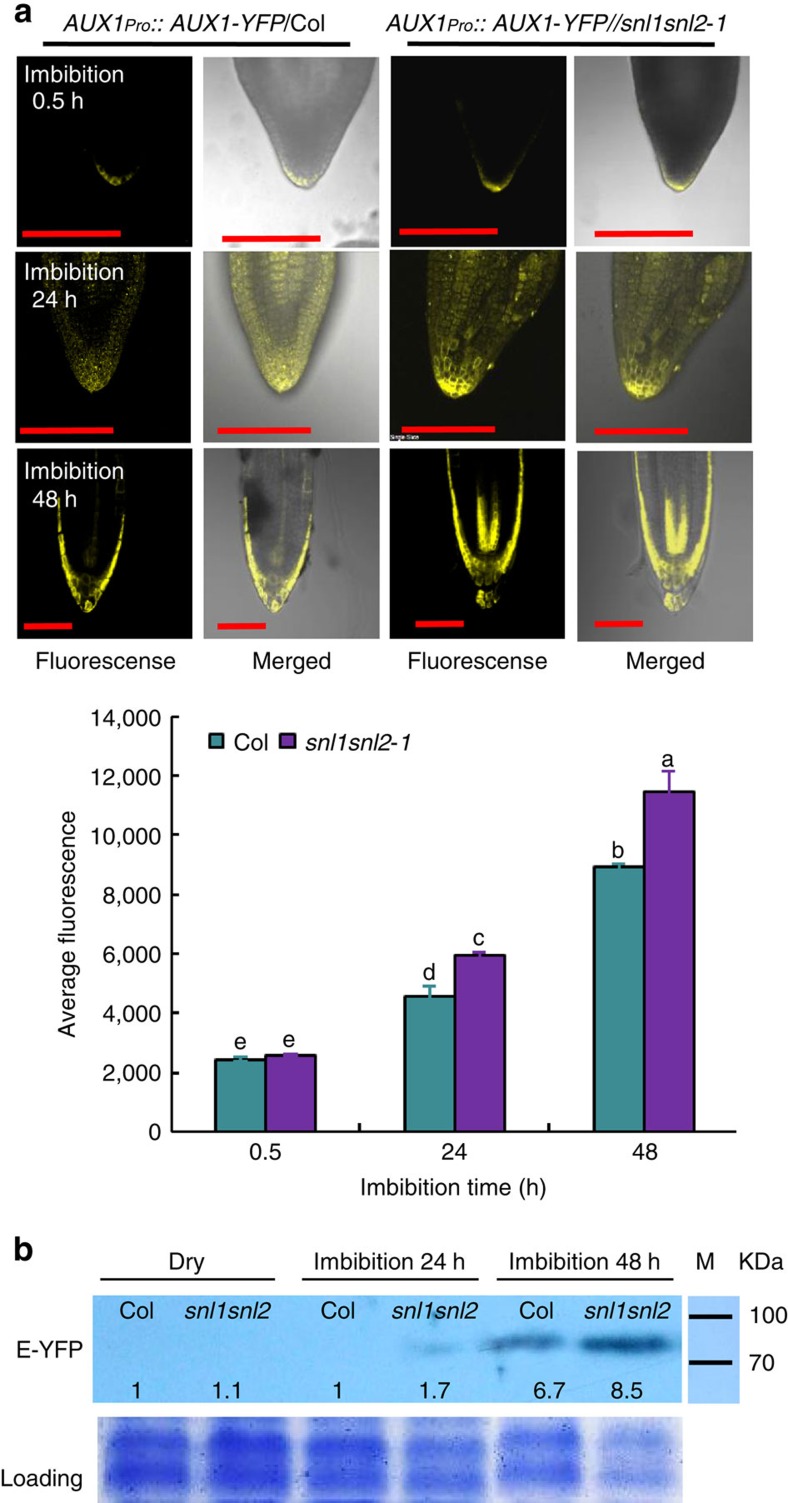
AUX1 protein accumulates in the radicles of *snl1snl2* embryos. (**a**) Upper panel, the AUX1-YFP protein was detected after 0.5, 24 and 48 h of seed imbibition in Col and *snl1snl2* radicles containing the *AUX1pro::AUX1-YFP* construct. Higher-fluorescence signals were detected in the *snl1snl2* mutant compared with the wild type. The lower panel shows a quantification of fluorescence signals by ImageJ software. The experiment was performed two times with independent plants, more than 15 embryos were observed and analysed statistically for each replicate and a representative result is shown. Different letters indicate a significant difference with Tukey's honestly significant difference test (*P*<0.05). Scale bars, 200 μm. (**b**) Immunoblot analysis of AUX1-YFP protein accumulation in dry and imbibed seeds from Col and *snl1snl2* plants containing the *AUX1pro::AUX1-YFP* construct. The E-YFP monoclonal antibody was used for detection, and Coomassie blue staining was used as loading control. Numbers indicate relative AUX1-YFP protein levels, normalized to the loading control. The experiment was performed three times, similar results were obtained and a representative result is shown.

We further studied the role of AUX1 in the enhanced germination speed of *snl1snl2* by introducing an *AUX1pro::AUX1-YFP* construct in this double mutant by crossing. The fluorescence signal from AUX1-YFP accumulated gradually during imbibition in both wild-type Col and *snl1snl2* mutant background. However, the double mutant showed a higher intensity of fluorescence than the wild type (Fig. 6a). Immunoblot analysis also showed a higher abundance of the AUX1-YFP protein in 24 and 48 h imbibed *snl1snl2* seeds compared with Col (Fig. 6b). This suggests that AUX1 has a role in the enhanced germination speed of the *snl1snl2* double mutant.

### *AUX1* is a target of histone deacetylation mediated by SNLs

**Figure 7:**
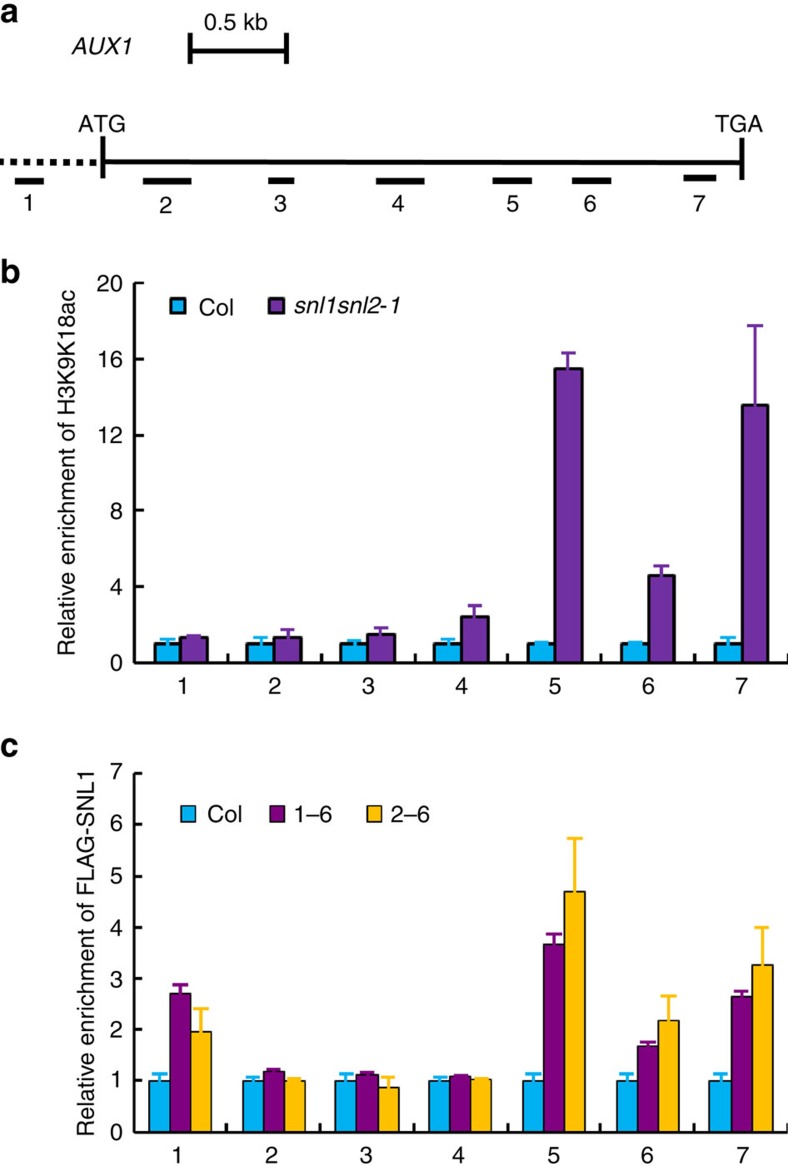
The *AUX1* gene has enhanced H3K9/K18 acetylation levels in *snl1snl2* seeds. (**a**) A schematic diagram of the *AUX1* gene structure. The dashed line indicates the ∼500 bp promoter sequence and the black line between the vertical dashes represents the open reading frame of the gene from start codon (ATG) to stop codon (TGA). The relative positions of the PCR-amplified fragments (1–7) for each tested region are depicted below the gene structure. Scale bar represents 500 bp. (**b**) ChIP analysis of H3K9/K18 acetylation levels on *AUX1*. Immunoprecipitates were obtained from 12 h imbibed seeds of Col and *snl1snl2* using H3K9/K18ac antibody. The accumulation of PCR product after immunoprecipitation has been normalized to *ACTIN8*. The bars indicate s.e.'s of three biological replicates. The numbers on the *x* axis represent the PCR-amplified sites described in **a**. (**c**) ChIP analysis of 3 × FLAG-SNL1 levels on *AUX1*. Immunoprecipitates were obtained from 7-day-old seedlings of Col and *35Spro::FLAG-SNL1/snl1snl2* lines (*1-6* and *2-6*) using a FLAG antibody. Relative amounts of the PCR products were calculated and normalized to *ACTIN8*, and the value in Col was set as one. The bars indicate s.e.'s of three biological replicates.

SNL1 and SNL2 function in histone H3K9/K18 deacetylation^32^. Therefore, *AUX1* could be a target of histone deacetylation mediated by SNL1 and SNL2. We analysed the acetylation levels of AUX1 in imbibed seeds of Col and *snl1snl2* by ChIP–qRT–PCR using H3K9K18ac antibody- and gene-specific primers (Supplementary Table 2). Enhanced acetylation levels were detected in the C-terminal gene-coding region of *AUX1* in *snl1snl2* (Fig. 7a,b) supporting previous reports that AUX1 is a target of H3K9 acetylation^40^. Several additional genes were analysed, and the *snl1snl2* double mutant showed increased acetylation at the promoter regions (region 1) of *PIN2*, and *CYP79B2* (Supplementary Fig. 7)^40^. To verify the direct interaction between SNL1 and auxin-related genes during histone deacetylation, we constitutively expressed 3 × *FLAG*-*SNL1* in the *snl1snl2* double mutant and obtained two homozygous lines, *1-6* and *2-6*. qRT–PCR and seed germination assays showed that a wild-type *SNL1* expression level and seed germination phenotype were recovered in the two transgenic lines (Supplementary Fig. 8). A ChIP–qRT–PCR analysis using anti-FLAG antibody indicated that 3 × FLAG-SNL1 proteins were mainly located in the C terminus of the *AUX1* gene (Fig. 7c), indicating that a potential interaction between SNL1 and *AUX1* occurred in the C terminus of the gene-coding region (Fig. 7c). Moreover, 3 × FLAG-SNL1 enrichment was also detected in the promoter region (region 1) of *PIN2*, and the promoter (region 1) and C-terminal gene-coding region (region 3) of *CYP79B2*, which is similar to the results from the H3K9K18ac ChIP assay (Supplementary Fig. 7). These data suggest that the upregulation of *AUX1* and other auxin-related genes in *snl1snl2* are tightly associated with increased histone H3K9K18 acetylation resulting from the loss of SNL1 and SNL2 function.

### CYCDs are regulated by AUX1 and SNLs

**Figure 8:**
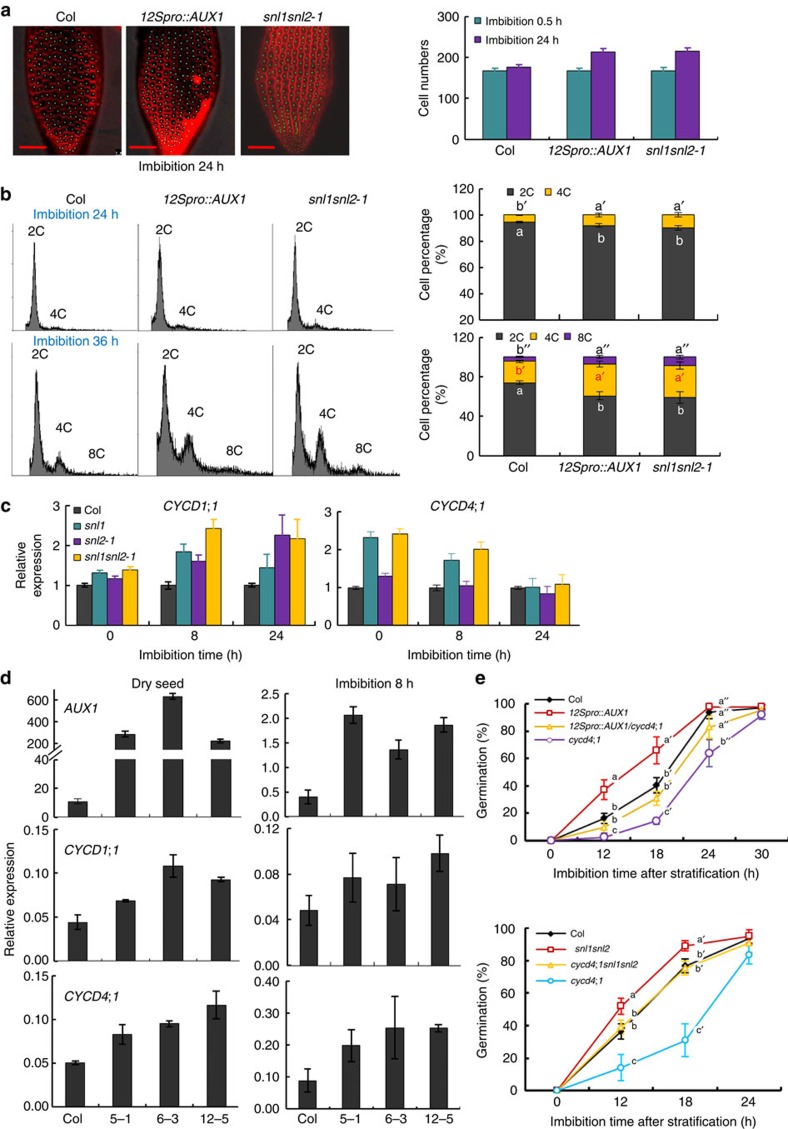
*AUX1* over-expressing lines and *snl1snl2* mutant show increased cell proliferation and *CYCD*s expression in the radicle during germination. (**a**) Cell numbers in the radicle of 24 h imbibed seeds from Col, *12Spro*::*AUX1* and *snl1snl2* were counted after FM4-64 (T-3166; Molecular Probes) staining and fluorescence microscopy imaging. The images in the left show stained radicles. The graphs in the right show endodermis cell numbers in Col, *12Spro*::*AUX1* and *snl1snl2* with s.d. (*n*>30). This experiment was performed two times with independent plants, similar results were obtained and a representative result is shown. Scale bars, 50 μm. (**b**) Changes in nuclear ploidy levels during seed imbibition. Histograms representing DNA content of embryo cells during seed imbibition. After-ripened seeds of Col, *12Spro*::*AUX1* and *snl1snl2* were imbibed for 24 h (upper panel) or 36 h (lower panel). 2C peak, G1 DNA content; 4C peak, G2 DNA content; 8C and 16C peaks, endoreduplicating cells. The graphs in the right show a quantification of the cell numbers at the different stages of the cell cycle based on the histograms. The bars indicate s.d. of three biological replicates and different letters indicate a significant difference determined by Tukey's honestly significant difference (HSD) test (*P*<0.05). (**c**) The relative expression of *CYCD1;1* and *CYCD4;1* in imbibed fully after-ripened seeds from Col and *snl* single and double mutants measured by qRT–PCR. Transcript levels were normalized to the level of A*CTIN8*. The relative transcript level in wild-type Col is set as one. The bars indicate s.e.'s of three biological replicates. (**d**) The relative expression of *AUX1*, *CYCD1;1* and *CYCD4*;*1* in fully after-ripened dry and imbibed seeds from Col and three *12Spro::AUX1* transgenic lines measured by qRT–PCR. Transcript levels were normalized to the level of A*CTIN8*. The bars indicate s.e.'s of three biological replicates. (**e**) Germination phenotypes of *12Spro::AUX1/cycd4;1* (top) and the triple mutant *cycd4;1snl1snl2-1* (bottom). Seeds were stratified by imbibition for 3 days at 4 °C before germination. Percentages of seed germination are means (±s.d.) based on the seeds from eight individual plants. Different letters at the same timepoint indicate a significant difference determined by Tukey's HSD test (*P*<0.05).

AUX1 localizes in the radicle tip of mature embryos^38^,^41^ and affects cell proliferation during embryogenesis in *Arabidopsis*^23^. To study the role of AUX1 in the promotion of radicle protrusion and growth, we counted the number of cells at the radicle tip in 24 h imbibed after-ripened seeds of Col, *12Spro::AUX1* and *snl1snl2*. We observed a higher number of radicle cells in *12Spro::AUX1* and *snl1snl2* compared with Col wild type (Fig. 8a). A flow cytometric analysis indicated enhanced levels of 4C and 8C nuclei in *12Spro::AUX1* and *snl1snl2* 24 and 36 h imbibed seeds compared with Col (Fig. 8b). This indicated faster cell cycling, which could contribute to the faster radicle protrusion of *12Spro::AUX1* and *snl1snl2* (Figs 1a and 5a). Cyclin D-type (*CYCD*) *1;1* and *CYCD4;1* are two key genes involved in cell cycling and seed germination^42^. Both of these genes showed enhanced transcript levels during imbibition of *snl1snl2* and *12Spro::AUX1* compared with Col wild type (Fig. 8c,d). It has been reported that cyclins could be regulated by phytohormones^43^. Our experiments indicated that a low auxin dose induced the expression of *CYCD1;1* and *CYCD4;1*, especially at early stages of imbibition (Supplementary Fig. 9). Moreover, loss of CYCD4;1 function in the *12Spro::AUX1* background reduced the germination speed of *12Spro::AUX1* back to the wild-type level. Similarly, knockout of CYCD4;1 in the *snl1snl2* mutant restored seed germination speed to wild type (Fig. 8e). These results suggest that *CYCD1;1* and *CYCD4;1* play an important role in radicle promotion and growth downstream of AUX1 and SNL1/SNL2.

## Discussion

*SNL1* and *SNL2* are highly expressed in dry seeds, but their transcript levels decrease during seed imbibition and germination (Supplementary Fig. 10)^32^ (http://bbc.botany.utoronto.ca/efp/cgi-bin/efpWeb.cgi?primaryGene=AT3G01320&modeInput=Absolute). Here we showed that SNL1 and SNL2 negatively regulate radicle protrusion and growth during seed germination after dormancy release (Fig. 1a–c). SNL proteins are components of the histone deacetylation complex^32^. Interestingly, loss-of-function mutants of another component of this complex, HDA19, showed a similar accelerated seed germination phenotype as the *snl1snl2* double mutant (Supplementary Fig. 11) suggesting that the SNL1/HDA19 complex controls seed germination via histone deacetylation.

The embryo plays an essential role in seed germination, both by activating its own growth potential and by transducing signals to weaken the endosperm. Activation of cell elongation is crucial for embryonic growth^44^,^45^. GAs have been suggested to play a key role in seed germination by increasing growth potential of the embryo and/or by weakening of the endosperm cap^46^,^47^,^48^. However, our previous work showed that SNL1 and SNL2 did not influence seed germination through the GA pathway^32^. A phenotypic analysis of mutants related to ABA hydrolysis and ethylene pathways suggested that these processes do also not play major roles in SNL1/SNL2 control of seed germination (Supplementary Fig. 12)^49^ although they are important in the regulation of seed dormancy in *snl1snl2* (refs 32, 49). Auxin has been shown to promote seed germination at low doses (Fig. 4)^16^,^17^. Therefore, the higher IAA accumulation in dry and imbibed seeds of *snl1snl2* (Fig. 3j) might explain the increase in germination rate. Pharmaceutical analyses further demonstrated that inhibitors of auxin synthesis and transport reduce seed germination rate of *snl* mutants to wild-type levels (Fig. 1d), supporting the role of auxin in seed germination. This suggests that auxin distribution controlled by transporters might have an important role in seed germination of *snl1snl2*.

SNL1 represses gene transcription by histone deacetylation^32^,^50^. The enhanced transcript levels of auxin-related genes in *snl1snl2* probably result from increased histone acetylation abundance. The ChIP assay with anti-H3K9/18ac and anti-FLAG confirmed this positive relationship (Fig. 7 and Supplementary Fig. 7). We hypothesized that one or more of the auxin-related genes could play a key role in the control of seed germination by SNL1 and SNL2. An analysis of a number of mutants and over-expression transgenic plants of several auxin-related genes, including *AUX1*, *PIN2*, *PIN3* and *ARF5* did not reveal seed germination phenotypes, except for *AUX1* (Fig. 5 and Supplementary Fig. 6). Moreover, loss of AUX1 function reduced the sensitivity of seed germination in response to exogenous auxin treatment at both low and high doses (Supplementary Figs 13 and 14). This suggests that AUX1 is a good candidate for the regulation of germination rate mediated by SNL1 and SNL2. Although loss of AUX1 function did not change germination speed (Supplementary Fig. 6b), *AUX1* over-expression resulted in a significant acceleration in seed germination (Fig. 5a–c). Previous studies showed that AUX1 levels increase within the root tip during radicle growth on seed germination^51^, which is consistent with our results (Fig. 6a). Moreover, we found that AUX1 levels in the root tip of *snl1snl2* are higher than that in the wild type during seed germination (Fig. 6). Genetic experiments showed that AUX1 is required for the accelerated germination speed of *snl1snl2* since the *snl1snl2aux1* triple mutant had a similar germination speed as wild-type seeds (Fig. 5d). Band *et al*.^52^ showed that the AUX1/LAX influx carriers are more important to create a proper distribution of auxin at the root tips of seedlings than efflux carriers such as PIN1 and PIN2. This is in agreement with our observations that germination speed depends more on AUX1 compared with the other auxin-related genes that we analysed. A possible explanation could be the more distinct functions of the *AUX1*/*LAX* genes^41^ in plant development compared with the redundant functions of most auxin efflux transport genes^53^.

Loss of function of AUX1 causes altered auxin distribution and can result in decreased auxin content^37^,^38^,^39^. We propose that AUX1 regulates radicle protrusion and growth by modifying auxin distribution and concentration. In support, both GC–MS measurements and assays with the auxin reporter DR5::GUS showed increased auxin levels in dry and early imbibed seeds of *12Spro::AUX1* transgenic lines, but lower levels in the *aux1-T* mutant compared with the wild type (Fig. 5e,f and Supplementary Fig. 15). Altogether our results indicate that AUX1 has an important but not essential role in radicle protrusion and early growth by regulating auxin content and distribution in the radicle tip downstream of SNL1 and SNL2. However, the mechanisms by which auxin promotes seed germination are not well understood. Auxin is known to influence plant development by controlling cell division and proliferation^34^,^35^. Previous studies have shown that activation of cell division is important to promote successful seed germination and early seedling growth in *Arabidopsis*^42^. We investigated cell proliferation during seed germination of *snl* mutants and *12Spro::AUX1* transgenic lines, and obtained four pieces of evidence indicating that cell division within the radicle tip is important for their increased germination speed. First, the radicle tip contains more cells in *12Spro::AUX1* lines and *snl1snl2* compared with wild type during seed imbibition (Fig. 8a). Second, a flow cytometric analysis showed that imbibed seeds of *12Spro::AUX1* and *snl1snl2* contained a higher percentage of polyploid embryo cells compared with wild type (Fig. 8b). Third, the cell division-related genes *CYCD1;1* and *CYCD4;1,* which encode D-type cyclins that act as rate limiting controllers of germination rate^42^, showed higher expression levels in the seeds of *12Spro::AUX1* and *snl1snl2* compared with wild type (Fig. 8c,d). Fourth, loss of CYCD4;1 function in *12Spro::AUX1* or *snl1snl2* background reduced seed germination speed to wild-type levels (Fig. 8e). These results indicated that the accelerated radicle protrusion and early growth of *snl1snl2* and lines over-expressing AUX1 could be at least partially caused by a higher cell mitosis or endocycle mediated by the D-type cyclins CYCD1;1 and CYCD4;1. Previous reports indicated that some *CYCDs* can be induced by auxin^43^. In accordance, we showed that exogenous application of low doses of IAA and 2,4-D increased *CYCD1;1* and *CYCD4;1* transcript levels in seeds (Supplementary Fig. 9). The radicle tips of *snl1snl2* and *12Spro::AUX1* lines accumulated relatively high levels of auxin compared with wild type as shown by a GC–MS assay and the auxin response reporter DR5::GUS (Figs 3 and 5e,f). This is likely to contribute to the enhanced expression of *CYCD1;1* and *CYCD4;1* transcripts in the radicles of both lines, causing accelerated radicle protrusion and growth.

**Figure 9:**
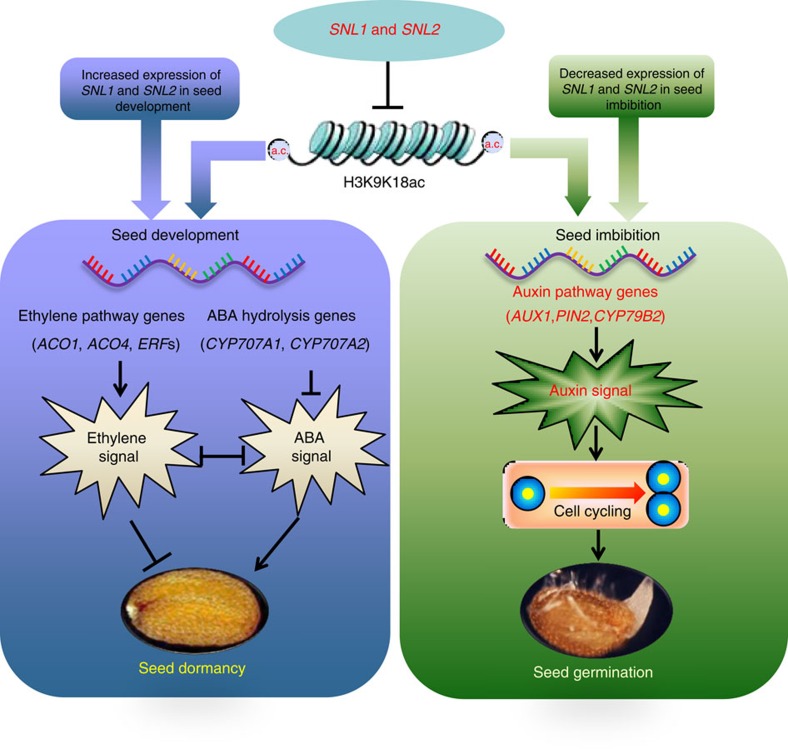
A hypothetical model for the regulation of seed dormancy and germination by SNL1 and SNL2. The expression of SNL1 and SNL2 increases gradually during embryo development and seed maturation causing a decrease in acetylation level of ABA hydrolysis genes (*CYP707A1* and *CYP707A2*) and some ethylene pathway genes (*ACO1* and *ACO4*). This promotes ABA signalling and represses ethylene signalling leading to the establishment of seed dormancy. During imbibition of after-ripened seeds, the expression of SNL1 and SNL2 declines, causing an increase in acetylation levels of auxin pathway genes (for example, *AUX1*). Subsequent activation of their transcription leads to increased auxin levels and signalling, followed by enhanced cell division promoting seed germination.

In this work we showed the role of SNL1 and SNL2 in germination speed, whereas we previously described the pathway by which SNL1 and SNL2 control seed dormancy establishment^32^. By combining these regulatory functions, we propose a complete model for the control of seed dormancy and germination by histone deacetylation associated with SNL1 and SNL2 (Fig. 9). During the embryo development and seed maturation the expression of SNL1 and SNL2 increased gradually, which decreased the acetylation level of ABA hydrolysis genes (*CYP707A1* and *CYP707A2*) and some ethylene pathway genes (*ACO1* and *ACO4*). This led to a promotion of ABA signals and a repression of ethylene signals, contributing to seed dormancy establishment. During imbibition and germination of after-ripened seeds, the expression of SNL1 and SNL2 declined, which increased the acetylation of auxin pathway genes (for example, *AUX1*) and activated their transcription. Subsequently, auxin levels and signalling increased, causing an activation of cell mitosis or endocycle to promote radicle growth and seed germination. We conclude that the complexes associated with SNL1 and SNL2 are important hubs for the establishment of seed dormancy and the regulation of germination in *Arabidopsis*.

## Methods

### Plant materials and growth conditions

All experiments were performed with *Arabidopsis thaliana* Columbia (Col) wild-type plants, or mutants in the Col background. T-DNA insertion lines Sail_1151_F09 (*snl1*), Salk_097168 (*snl2-1*), Salk_073 549 (*snl2-2*) and GK_344D08 (*cycd4;1*) were previously described^32^,^42^, GK_900B12 (*snl1-2*) was ordered from NASC (University of Nottingham) and homozygous lines were identified by genome PCR using primers 900B12LP/RP and a T-DNA primer (Supplementary Table 2). Mutants of the auxin pathway genes, *aux1-21*, *aux1-22*, *aux1-T*, *pin2* (*eir1-1*), *pin3-3* and *pin3-5* have been described before^37^,^54^,^55^. Other T-DNA mutants associated with the auxin pathway (for example, *YUC3*, *CYP79B*2 and *CYP79B3*) were ordered from ABRC (Ohio State University), identified by genome PCR and analysed for their seed germination phenotypes. *DR5::GUS* and *AUX1pro::AUX1-YFP* lines have been previously described^56^,^57^ and were individually crossed into *snl1snl2*. The double mutant *snl1-2snl2-1* and the triple mutants *snl1snl2-1aux1-21* and *cycd4;1snl1snl2* were produced by crossing, as well as the *12Spro::AUX1/cycd4;1*. Homozygous plants were isolated from F2 populations and F3 plants or further generations were used for analysis.

Seeds were sown in soil and grown in the greenhouse under photoperiodic cycles of 16 h light and 8 h dark at 22 °C. Seeds were sown on half-strength Murashige and Skoog (MS) medium after sterilization with 10% (v/v) NaClO. Plates were kept in the dark at 4 °C for 3 days to break dormancy (stratification) before moving into a climate chamber with a photoperiod of 16 h light and 8 h dark at 22 °C.

### Germination tests

Germination tests were performed as described by Alonso-Blanco *et al*.^58^ Radicle protrusion was regarded as seed germination completion. All germination experiments were performed on filter paper in 6 cm Petri dishes. Each germination experiment was performed with at least eight replicates (consisting of 80–100 seeds from one individual plant per Petri dish) per genotype. The average germination ratio was determined in a climate room (25 °C, 16 h light with 80–90 μmol m^−2^ s^−1^ light intensity). The radicle growth and seed germination speed were determined at different time points during imbibition (for example, 12, 18, 24 and 48 h). For AVG (A6685; Sigma-Aldrich), TIBA (T5910; Sigma-Aldrich), NOA (255416; Sigma-Aldrich), IAA (I2886; Sigma-Aldrich) and 2,4-D (76514; Sigma-Aldrich) treatments, seeds were imbibed in Tris-HCl (10 mM) buffer supplemented with or without (Mock: 10 mM Tris-HCl buffer, pH 7.5) freshly prepared various concentrations of the reagents. Germination assays with seeds from different genotypes were performed with plants grown simultaneously in the same tray and stored under identical conditions. All the seed samples used were non-dormant and stored for at least 4 months at room temperature. For some experiments, seeds were given a 3 days stratification treatment at 4 °C before the germination test.

### Constructs and plant transformation

Total RNA was isolated from young Col leaves using the Qiagen RNeasy kit (Qiagen). cDNAs were generated by SuperScript II reverse transcriptase (Invitrogen). The open reading frame of *AUX1* was amplified using Phusion DNA Polymerase (NEB) with primers AUX1-F/-R (Supplementary Table 2) and introduced into a Gateway Entry vector (Invitrogen). The Gateway Entry clones of *ATR4*, *ARF5*, *CYP79B2* and *YUC3* were ordered from NASC and destination clones were constructed by inserting cDNAs into the 12S::pLEELA vector, which is a derivative of pLEELA. The 12S::pLEELA vector was constructed by replacing the 35S promoter with the 12S promoter in pLEELA. *CRUCIFERIN 3* (At4g28520) encodes a 12S seed storage protein and expresses specifically and highly in dry seeds^59^,^60^. The 12S promoter (1.85 kb) was amplified with the primer pair 12S-F and 12S-R from the *CRUCIFERIN 3* promoter region (Supplementary Table 2). The fusion genes were transformed in Col by *Agrobacterium tumefaciens* strain GV3101 pm90RK using the floral dip method^61^. Transformants were selected based on their ability to survive after 7 days in MS medium with 10 mg l^−1^ DL-phosphinothricin. The 3:1 segregating transformant lines were selected on MS medium with 10 mg l^−1^ DL-phosphinothricin. T3 homozygous transgenic plants were used for phenotypic analyses. The *35Spro::3* × *FLAG-SNL1* was constructed and transformed into the *snl1snl2* mutant to obtain the complementing line *35Spro:: 3* × *FLAG-SNL1/snl1snl2-1*. All of the constructs used in this study were confirmed by sequencing.

### GUS staining and fluorescence visualization

Three independent transgenic lines were used for GUS staining using 5-bromo-4-chloro-3-indolyl-β-D-glucuronic acid (X-Gluc, X8060; Amresco) as substrate^62^. Mature after-ripened embryos were soaked and imbibed in dH_2_O or IAA solution dissolved in Tris-HCl (10 mM) buffer for the indicated periods. Seed coats were removed under a dissecting microscope using a fine forceps and a 250 mm tungsten tip needle. Embryos dissected from the seed coat or seedlings were transferred to 1.5 ml Eppendorf tubes containing GUS staining buffer. After staining, embryos and seedlings were soaked in 95 and 75% ethanol and thereafter examined and imaged under a stereoscope and Nikon 80i Upright microscope (Nikon)^62^. The radicle length was measured using the NIS-Elements D software and GUS density was analysed by ImageJ software^63^. Visualization of AUX1-YFP was conducted using a Zeiss confocal microscope (LSM 510 META) (excitation wavelength 480±20 nm, emission wavelength 510±20 nm). Zeiss LSM Image Browser software (version 3.2.0.70) was used for image acquisition.

### Radicle cell number statistics

The dissected embryos were stained in FM4-64 (T-3166; Molecular Probes) at a final concentration of 10 μm to visualize the cells with a Zeiss confocal microscope (LSM 510 META). The part from the tip to the middle of the radicle (1/2 radicle length) was used for cell counting and statistically analysed. Only endodermis cells were captured and counted since the endodermis cells in the radicle tip differentiated earlier and stained better than other cells, which facilitated fluorescence signal observation and accurate statistics. Green spots were artificially added to the photo in cells to enable precise counting using Photoshop software. For each line, at least 30 embryos were stained and statistically analysed.

### Protein extraction and immunoblotting

A unit of 20 mg dry or imbibed seeds were ground in a mortar with liquid nitrogen, after which protein was extracted with a buffer containing 6 M urea, 2 M thiourea, 0.2%(v/v) Triton X-100, 0.2%(w/v) sarcosyl and 2 mM dithiothreitol in 100 mM Tris-Cl, pH 7.5. After two cycles of 30 min shaking the supernatant was collected by centrifugation at 4 °C for use^64^. Protein concentration was determined by Bradford dye reagent (Bio-Rad) using BSA as a standard. A unit of 50 μg proteins were separated by electrophoresis on a 4–10% polyacrylamide gel and transferred to Immobilon-P membrane (Amersham) for immunoblotting. Traditional semi-dry transfer was performed using the TE77XP blot module (Hoefer). Protein accumulation was detected using anti-E-YFP monoclonal antibody (1: 1,000 dilutions; AB124-01, Toyephon) and visualized with X-film using a horseradish peroxidase-conjugated anti-mouse IgG secondary antibody (1: 5,000 dilutions; 330, MBL) and the Super ECL plus detection system (Applygen). The relative signal density was analysed by ImageJ software, the signal in dry Col seeds was set as one. Uncropped scans of all western blots and DNA gels are shown in Supplementary Fig. 16.

### RNA extraction and qRT–PCR

Total RNA from seedlings or seeds was extracted using the Qiagen RNeasy kit and RNAqueous small-scale phenol-free total RNA isolation kit (Ambion) according to the manufacturer's instructions and reverse transcribed using the SuperScript RT–PCR system (Invitrogen). One microgram of total RNA was used for reverse transcription and semi-quantitative PCR, which was performed at least four times for each sample using rTaq polymerase (TaKaRa). *ACTIN8* (*ACT8*) was amplified as a control. The linear range of detection for each transcript was monitored and samples were run with 35 cycles for *AUX1* using primer pair AUX1-QF and AUX1-QR, and 30 cycles for *ACT8*. The qRT–PCR was performed using the KAPA SYBR FAST qPCR Kits (KAPA). The expression value for each gene was quantified using a standard curve with a serial dilution of plasmid of known concentration and normalized to the value of *ACT8* (ref. 65). At least three biological replicates were analysed. Primers are listed in Supplementary Table 2. Mean values and standard errors were calculated from three biological replicates.

### Measurement of free IAA

After-ripened seeds of Col, *snl1*, *snl2-1*, *snl1snl2* mutants and *12Spro::AUX1* transgenic plants were used for the measurement of free IAA levels. The extraction, purification of samples and analysis of free IAA by GC–MS were performed according to the method described by Edlund *et al*.^66^, except that an Agilent 7000B GC-MS Triple Quad GC–MS was used with the separation performed in a DB-5ht column (Agilent). The internal standard [^13^C_6_] IAA was purchased from Cambridge Isotope Laboratories (http://www.isotope.com).

### ChIP assay

About 1.5 g 12 h imbibed seeds or 7-day-old seedlings were used for ChIP assay. Chromatin preparation and immunoprecipitation were performed as described^67^. Imbibed seeds or seedlings were fixed in 1% formaldehyde for 10 min in a vacuum. Glycine was added to a final concentration of 0.125 M, and the reaction was terminated by incubation for 5 min in a vacuum. Seedlings were rinsed three times with distilled water and frozen in liquid nitrogen. After isolation, chromatin was sheared to 500–2,000 bp fragments by sonication (Branson Sonifier 250). Immunoprecipitation was performed by adding specific antibody and protein G agarose/salmon sperm DNA (Millipore) to the extract. ChIP assays were performed using 4 μg anti-H3K9/18ac (07–593; Upstate) and anti-FLAG M2 (F1804; Sigma-Aldrich) antibodies. After washing, immune complexes were eluted from the protein G beads and reverse crosslinked by incubation at 65 °C over night. Samples were treated with proteinase K for 1 h at 65 °C and RNase A for 1 h at 37 °C. DNA was extracted in a final volume of 40 μl using the QIAquick PCR purification kit (Qiagen). A volume of 0.5 μl DNA was used for each qRT–PCR. The qRT–PCR with SYBR Premix Ex Taq (TaKaRa) was carried out with a real-time system (Eppendorf). Each sample was assayed in triplicate by PCR. *ACT8* was used as internal control in the ChIP assay^32^,^65^. Primers used for ChIP assays are listed in Supplementary Table 2 online.

### Flow cytometry

Seed samples were collected at different stages of germination and more than 150 mature embryos were isolated from their seed coats. Nuclei were released by chopping and analysed with the Moflo XDP Cell Sorter (Beckman-Coulter) as described^68^. The data were analysed by Summit software and statistical significance analyses were made.

### Statistical analysis

For physiological and biochemical data, an analysis of variance was performed to investigate whether there was a significant difference between the samples. If a significant difference was found, a Tukey's honestly significant difference test was performed to determine which samples were responsible for the significant differences.

### Data availability

The authors declare that all data supporting the findings of this study are available within the paper and its Supplementary Information files or are available from the corresponding author on request.

## Additional information

**How to cite this article**: Wang, Z. *et al*. *Arabidopsis* seed germination speed is controlled by SNL histone deacetylase-binding factor-mediated regulation of *AUX1*. *Nat. Commun.* **7**, 13412 doi: 10.1038/ncomms13412 (2016).

**Publisher's note:** Springer Nature remains neutral with regard to jurisdictional claims in published maps and institutional affiliations.

## Supplementary Material

Supplementary Information Supplementary Figures 1-16 and Supplementary Tables 1-2
