# Supplementary material for: Arabidopsis seed germination speed is controlled by SNL histone deacetylase-binding factor-mediated regulation of AUX1
Source: Nat Commun. 2016 Nov 11;7:13412. doi: 10.1038/ncomms13412 (PMC5114640; doi:10.1038/ncomms13412)
Supplement: Supplementary Information — Supplementary Figures 1-16 and Supplementary Tables 1-2 [file ncomms13412-s1.pdf]

## Supplementary Figures

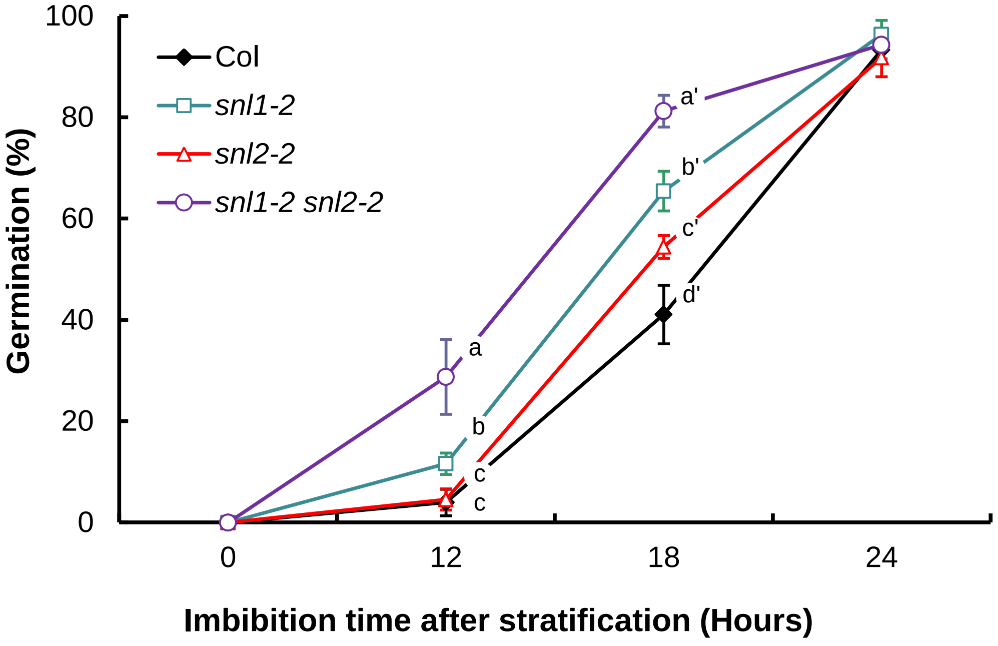


**Supplementary Figure 1. Seed germination phenotypes of *snl1-2, snl2-2* and *snl1-2snl2-2* mutants.** Germination phenotypes of wild type Col, *snl1-2, snl2-2* and *snl1-2snl2-2* mutants were determined in after-ripened seeds. Seeds were stratified for 3 days at 4 $^{o}$C. Percentages of seed germination are means (± SD) based on seeds from eight individual plants. Different letters at the same timepoint indicate a significant difference determined by Tukey's HSD test (*p*<0.05). SD, standard deviation.

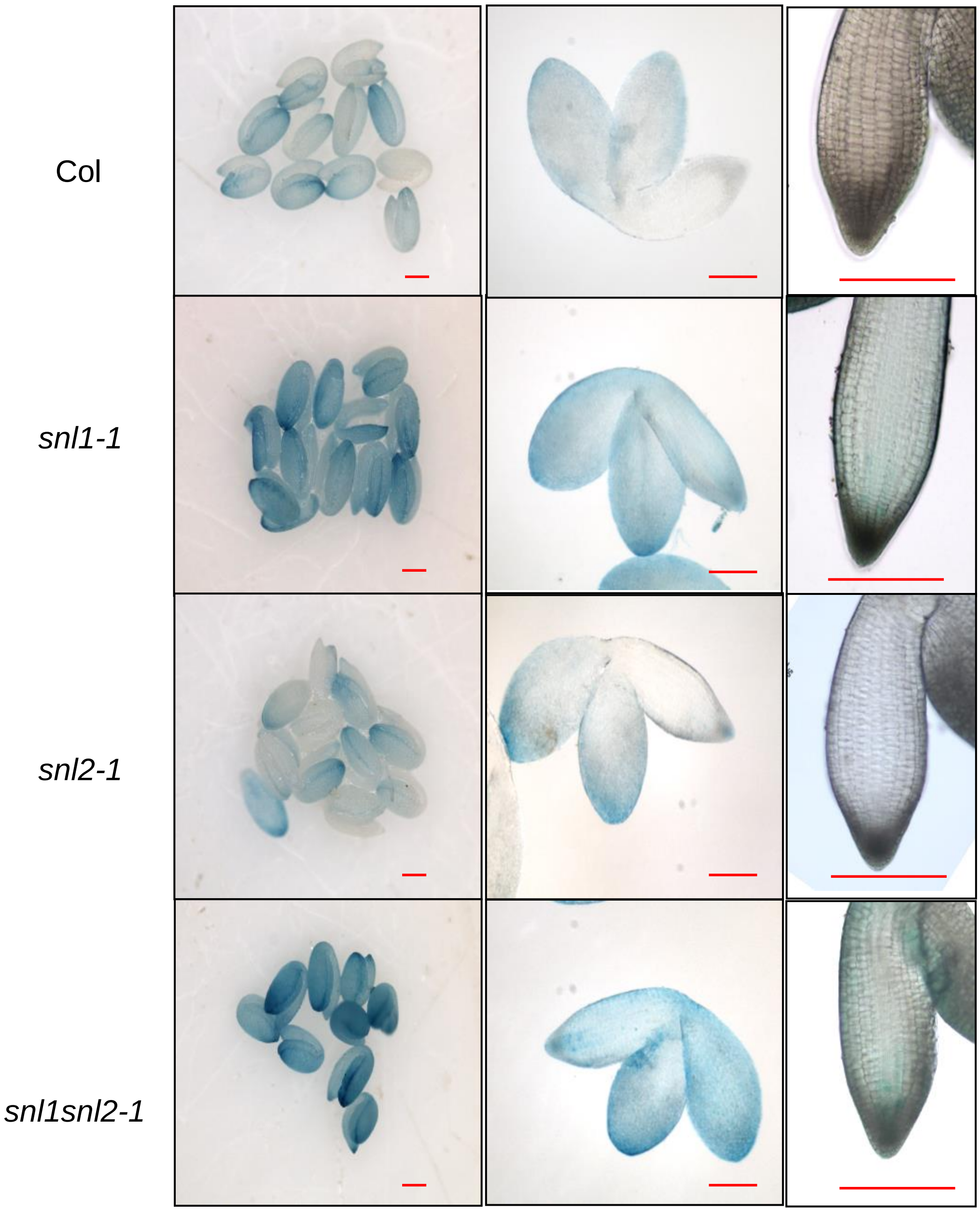


**Supplementary Figure 2. DR5::GUS levels in mature embryos of Col and *snl* mutants.**
The *DR5::GUS* construct was introduced into *snl* mutants by crossing. The images show GUS stained embryos from 0.5 hour imbibed after-ripened seeds. The experiment was performed with three independent lines and representative results are shown. Bar = 200 μm.

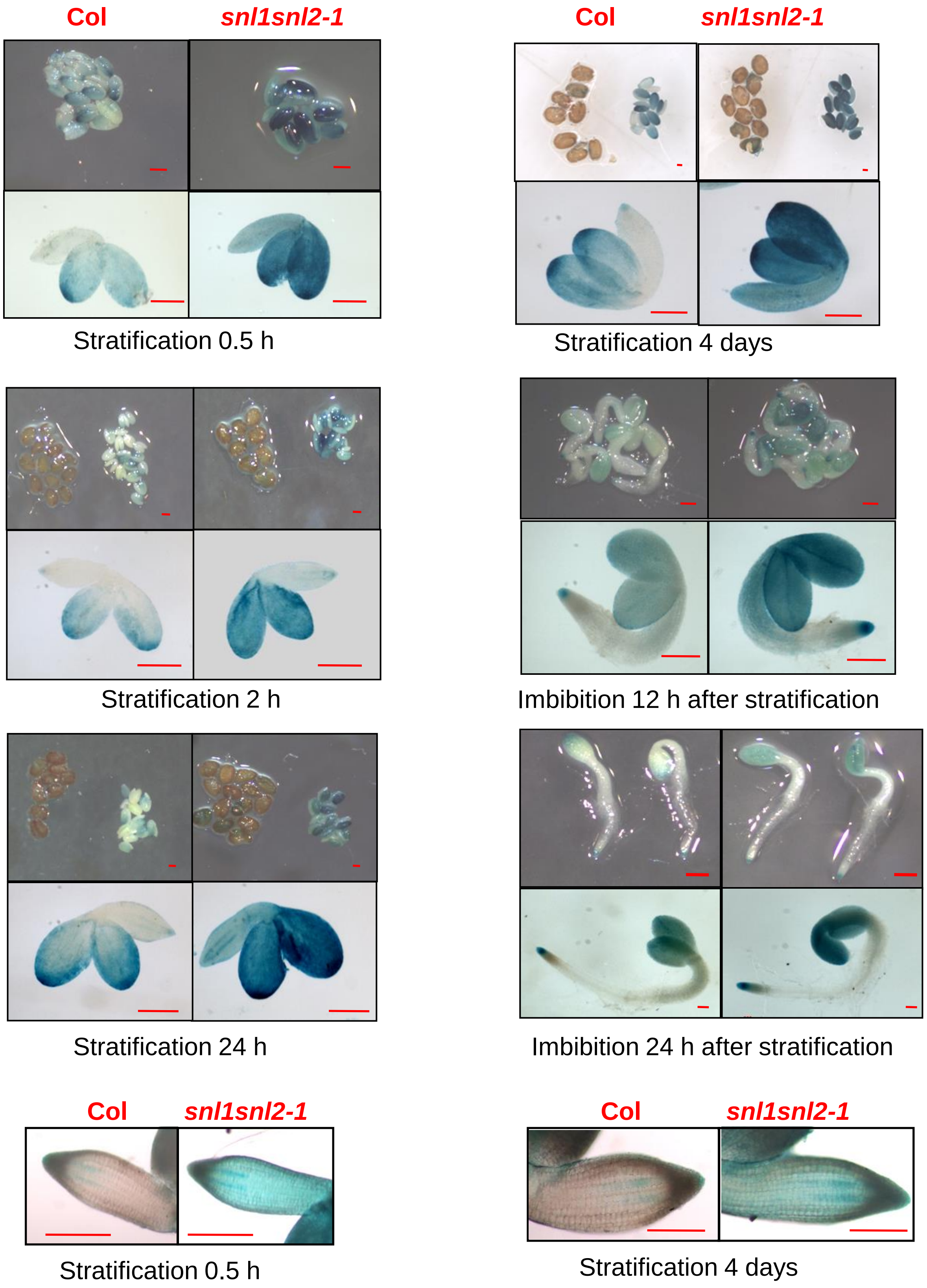


**Supplementary Figure 3. DR5::GUS levels in Col and *snl1snl2* during imbibition of stratified seeds.** The images show GUS stained seeds and embryos from 0.5 hour – 4 days stratified seeds. The experiment was performed with three independent lines and representative results are shown. Bar = 200 µm.

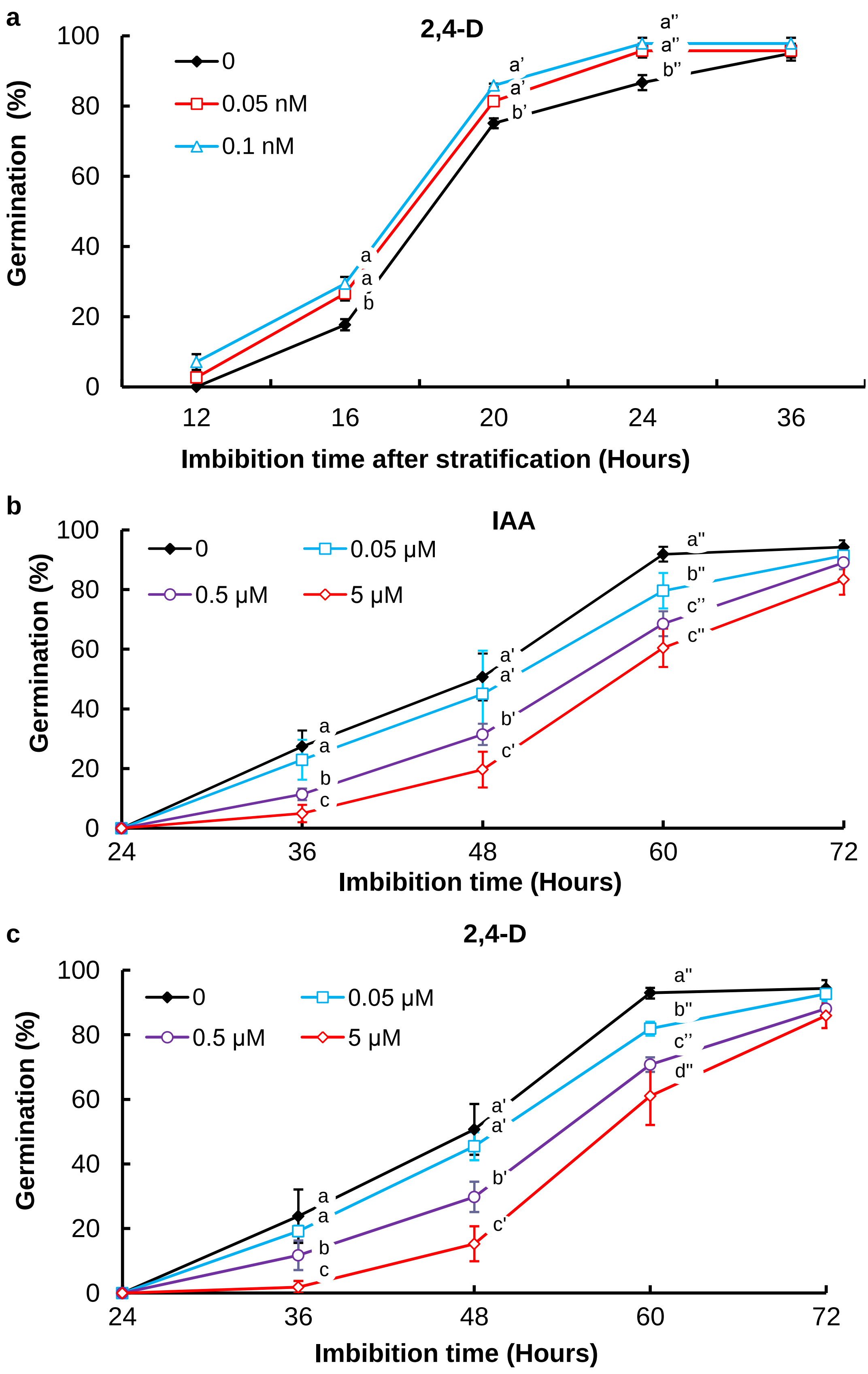


**Supplementary Figure 4. Auxin promotes or represses seed germination depending on its concentration.**

(**a**) Exogenous application of 2,4-D at low concentrations (0.05 and 1 nM) promotes germination of stratified Col seeds. The bars indicate standard deviation of eight biological replicates. Different letters at the same timepoint indicate a significant difference determined by Tukey's HSD test ($p$<0.05).

(**b**-**c**) Exogenous application of IAA (**b**) and 2,4-D (**c**) at high concentrations (0.05 μM-5 μM) represses germination of after-ripened seeds. The bars indicate standard deviation of eight biological replicates. Different letters at the same timepoint indicate a significant difference determined by Tukey's HSD test ($p$<0.05).

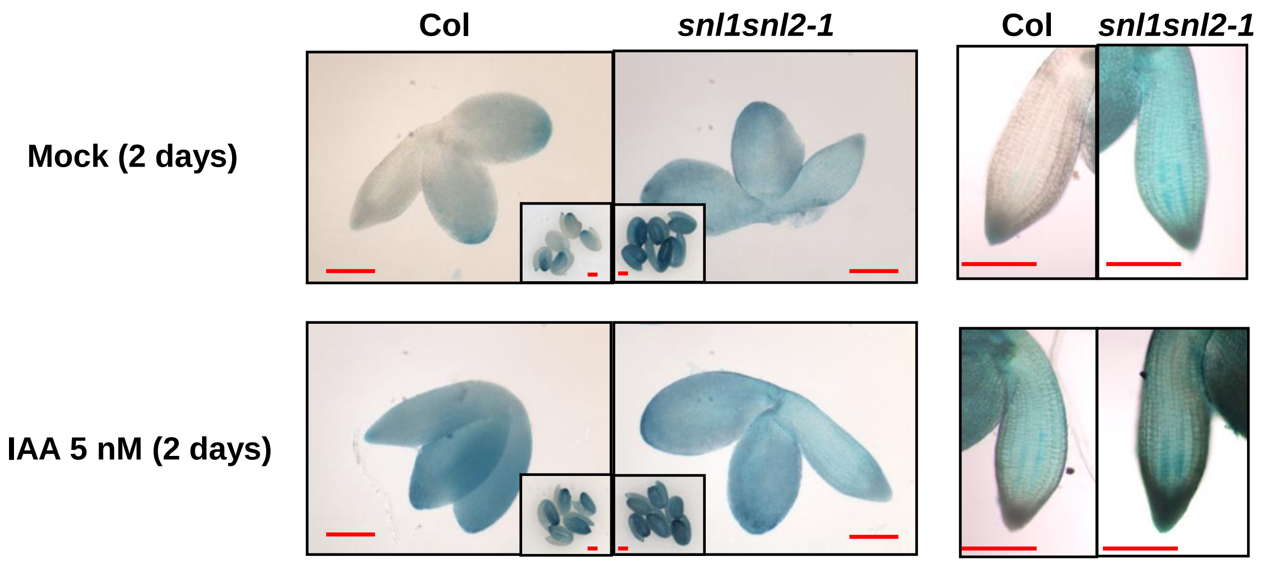


**Supplementary Figure 5. The response of DR5::GUS levels to IAA treatment in *DR5::GUS*/Col and *DR5::GUS/snl1snl2* lines.**

The images show GUS stained embryos from *DR5::GUS/Col* and *DR5::GUS/snl1snl2* seeds imbibed in 5 nM IAA for two days at 4 ºC. The experiment was performed twice with independent samples and representative results are shown. Bar = 200 µm.

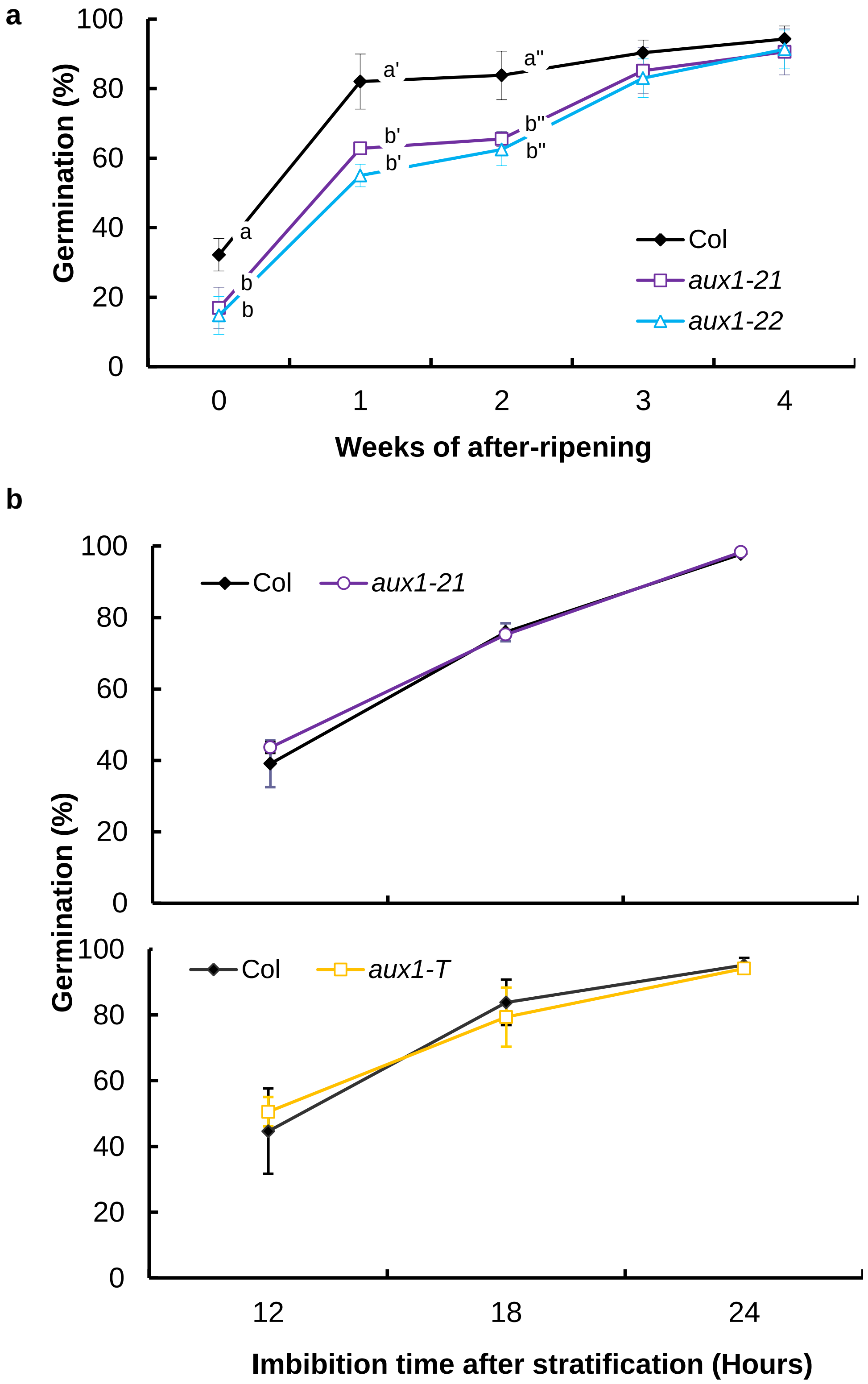


**Supplementary Figure 6. Seed germination phenotypes of *aux1* mutants.**
(**a**) Germination of Col, *aux1-21* and *aux1-22* seeds during after-ripening. Different letters at the same timepoint indicate a significant difference determined by Tukey's HSD test ($p$<0.05).
(**b**) Germination during imbibition of Col, *aux1-21* and *aux1-T* seeds that were stratified for 3 days. The bars indicate standard deviation of eight biological replicates.

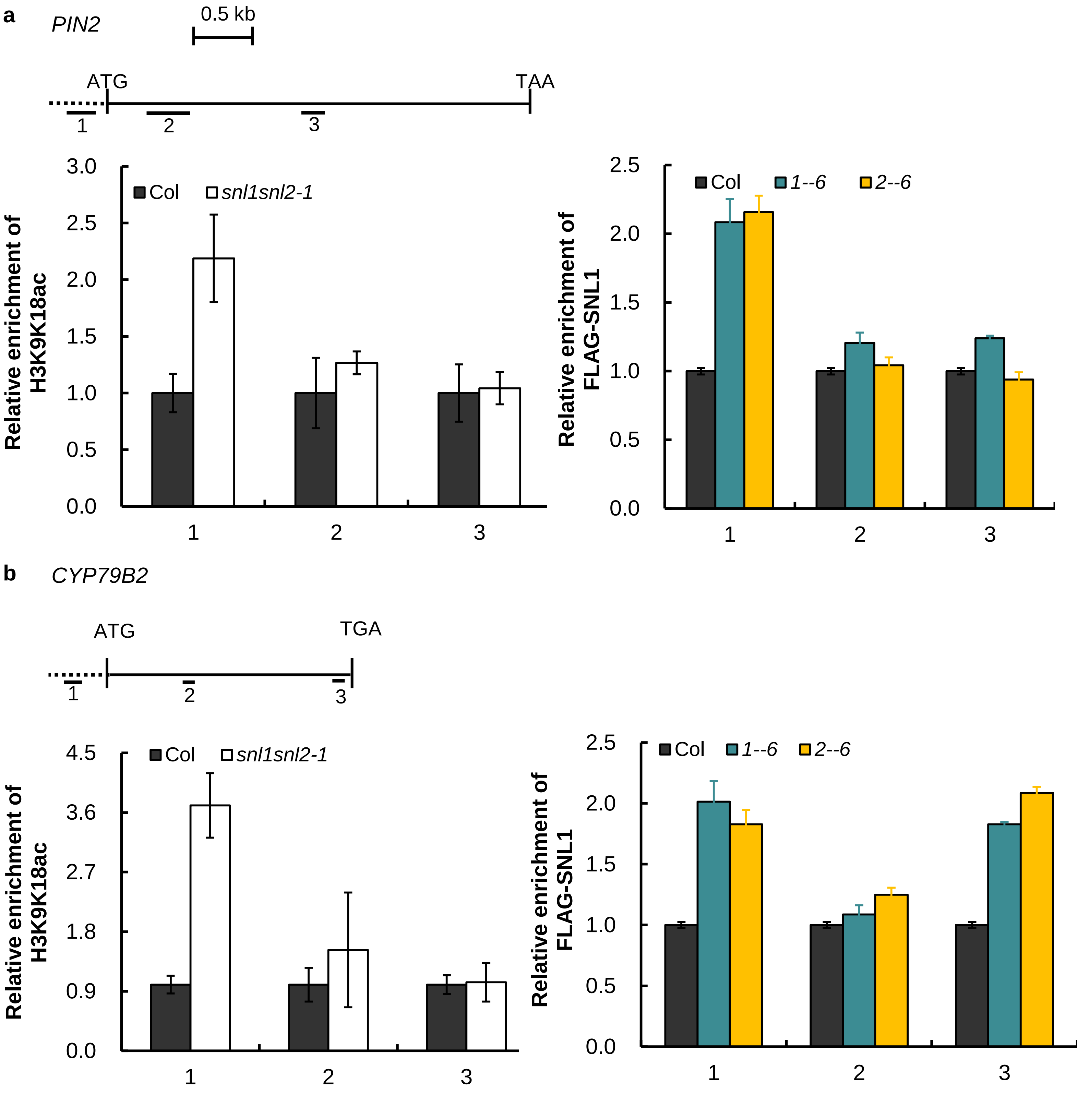


**Supplementary Figure 7. ChIP analysis of H3K9/K18 acetylation levels and SNL1 occupancy at auxin related genes in Col, *snl1snl2* and *35Spro::3×FLAG-SNL1/snl1snl2*.**

Schematic diagrams of the *PIN2* (**a**) and *CYP79B2* (**b**) genes are shown in the top panel. Dashed lines indicate the ～500 bp promoter sequence. The black line between the vertical dashes represents the open reading frame of the gene from start codon (ATG) to stop codon (TAA/TGA). The relative positions of the PCR-amplified fragments (1-3) for each tested region are depicted below the gene structure. Scale bar represents 500 bp. The figures show the accumulated abundance of the three different regions in each gene after qRT-PCR with specific primers (Supplementary Table 2) using immunoprecipitates obtained from 16 h imbibed seeds of Col and *snl1snl2* using H3K9,18ac specific antibody (left) or from 7 days old seedlings of Col and *35Spro::FLAG-SNL1/snl1snl2-1* using FLAG antibody (right). Relative amounts of the PCR products were calculated and normalized to *ACTIN8*. The value of Col was set as one. The bars indicate standard error of eight biological replicates.

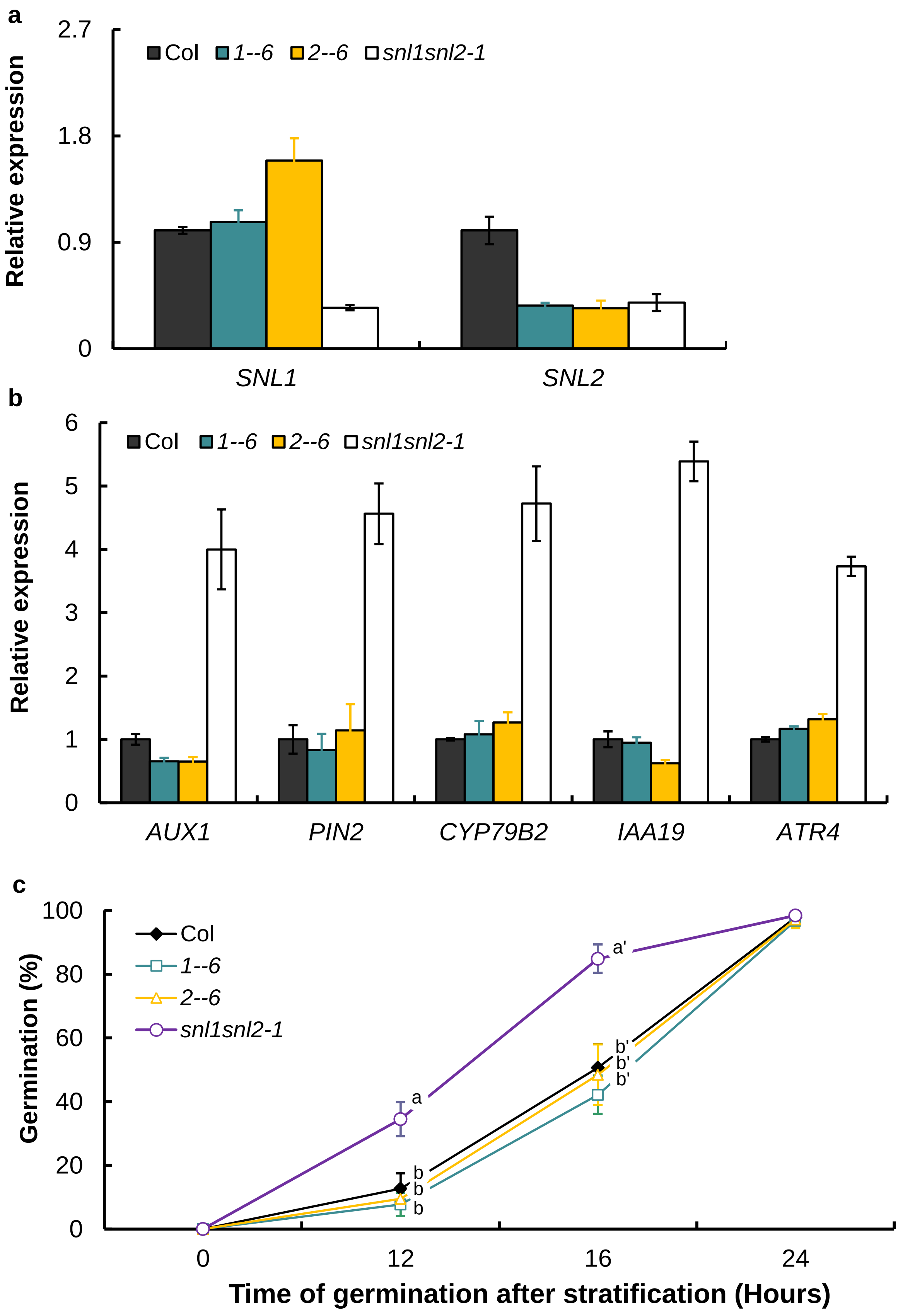


**Supplementary Figure 8. Transcript levels of *SNL*s and auxin related genes, and seed germination of *35Spro::3×FLAG-SNL1/snl1snl2-1* lines.**

(**a, b**) The relative expression of *SNL1* and *SNL2* (**a**) and auxin related genes (**b**) was measured by qRT-PCR in Col, *35Spro::FLAG-SNL1/snl1snl2* (*1-6* and *2-6*) and *snl1snl2* seeds. Transcript levels were normalised using the expression level of *ACTIN8* as an internal standard. The relative transcript level in Col is set at one. The bars indicate standard error of three biological replicates. Used primers are listed in Supplementary Table 2.

(**c**) Germination during imbibition of seeds from Col, *snl1snl2* and *35Spro::FLAG-SNL1/snl1snl2* lines *1-6* and *2-6* after stratification. The bars indicate standard deviation of eight biological replicates. Different letters at the same timepoint indicate a significant difference determined by Tukey's HSD test ($p<0.05$).

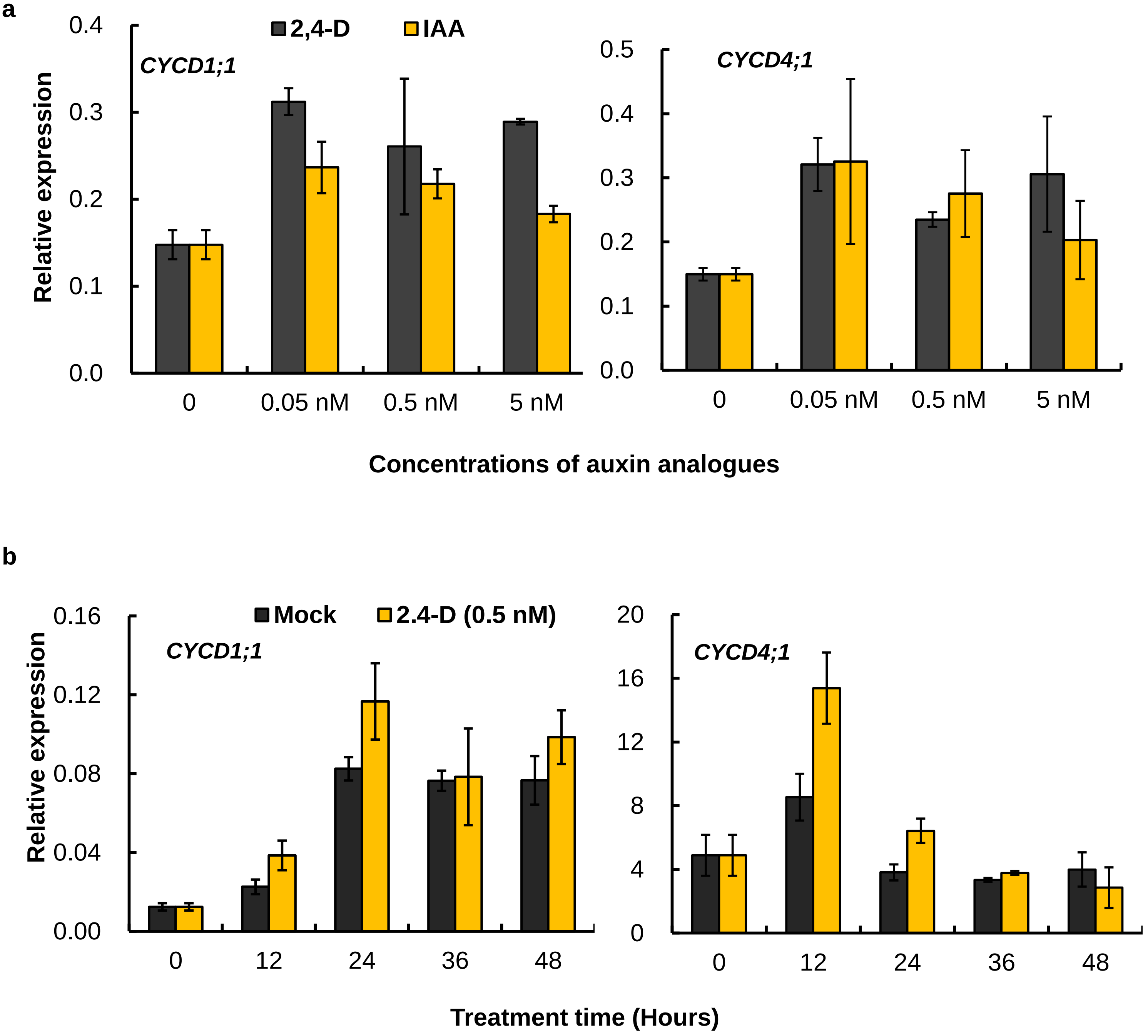


**Supplementary Figure 9. Auxin enhances expression of *CYCD1;1* and *CYCD4;1* in seeds.**
(**a**) Transcript levels of *CYCD1;1* and *CYCD4;1* are enhanced in seeds imbibed 24 h in low concentrations of 2,4-D and IAA (0.05 - 5 nM).
(**b**) The expression patterns of *CYCD1;1* and *CYCD4;1* in response to low concentration 2,4-D treatments (0.5 nM) and mock during 0 - 48 h seed imbibition. Transcript levels were determined by qRT-PCR and normalized with *ACT8*, used primers are listed in Supplementary Table 2. The bars indicate SE of three biological repeats.

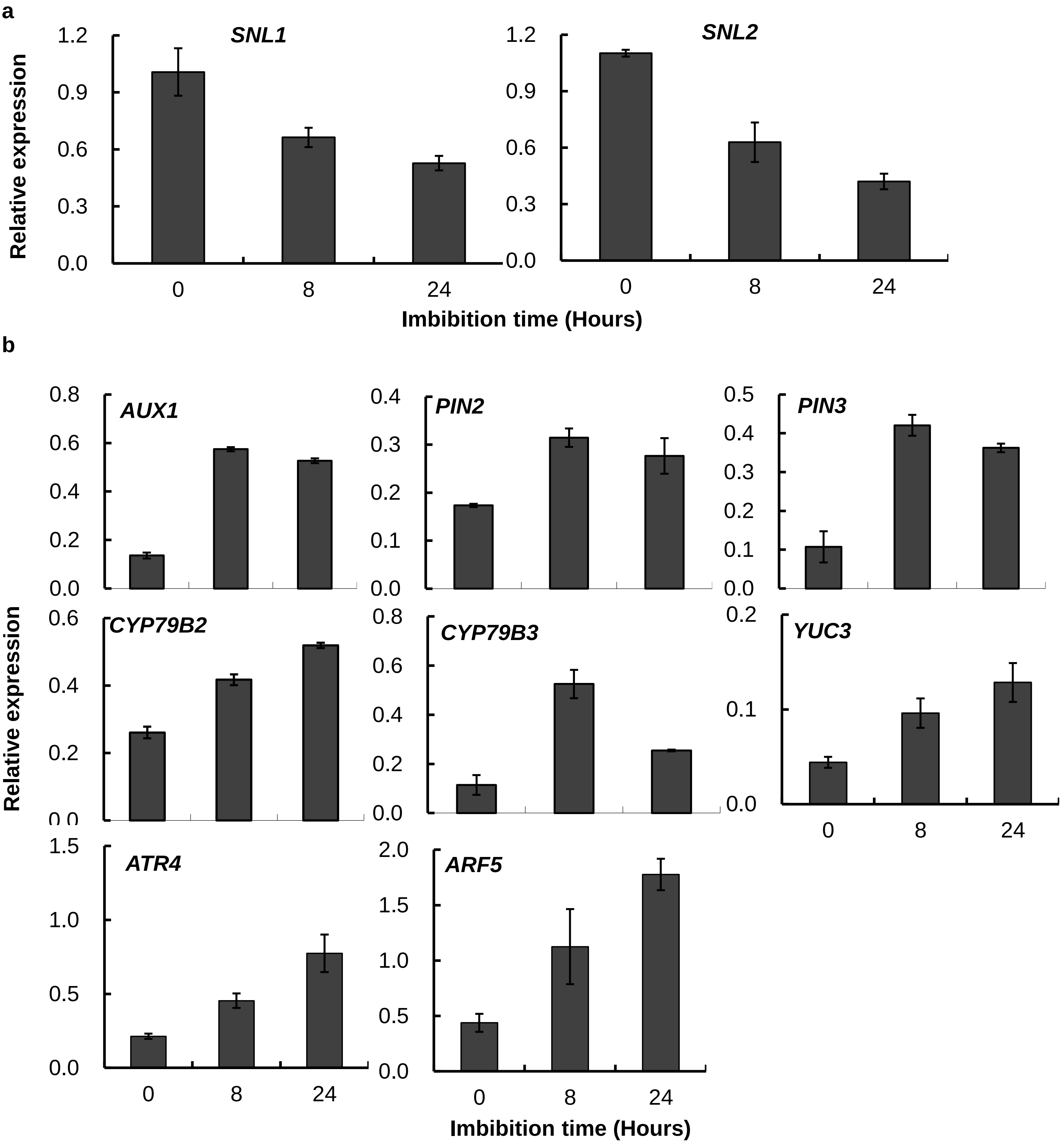


**Supplementary Figure 10. Expression profiles of *SNL*s and auxin pathway genes during seed imbibition.**

The relative expression of *SNL1*, *SNL2* (**a**) and auxin pathway related genes (**b**) was measured by qRT-PCR and normalized with *ACT8* during seed imbibition. The bars indicate SE of three biological repeats. The gene specific primers are listed in Supplementary Table 2.

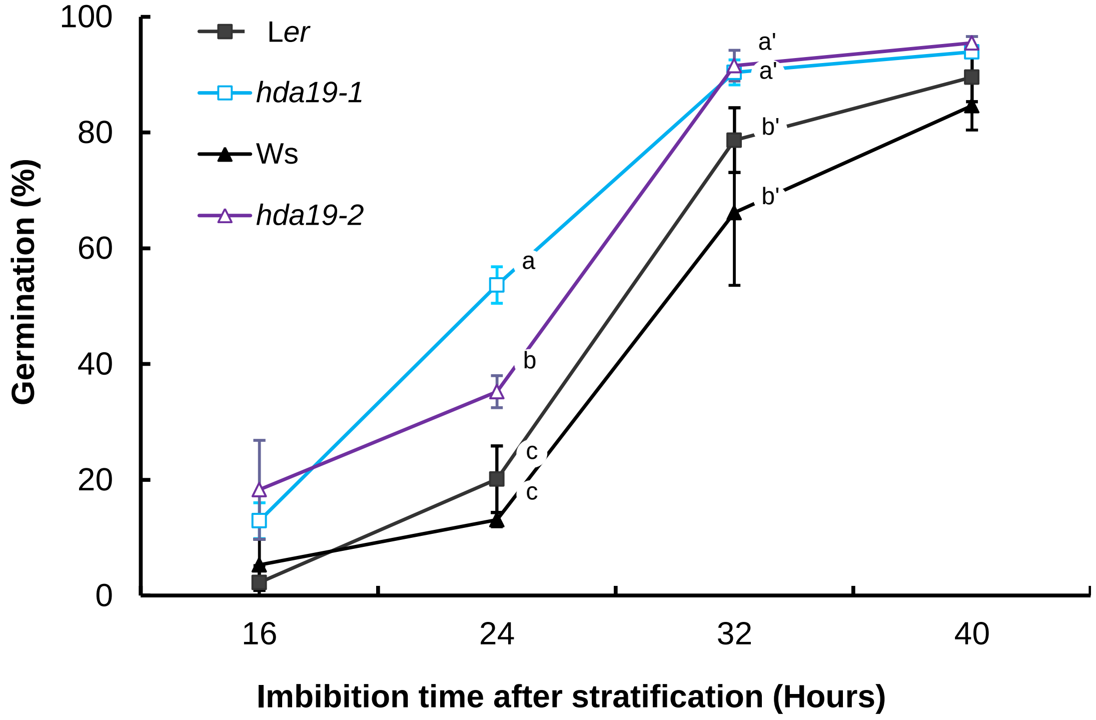


**Supplementary Figure 11. Germination phenotypes of *hda19* mutants.**
Germination during imbibition of seeds from *hda19-1* and *hda19-2* mutants and their corresponding wild-types L*er* and Ws after stratification. The bars indicate standard deviation of eight biological replicates. Different letters at the same timepoint indicate a significant difference determined by Tukey's HSD test ($p<0.05$).

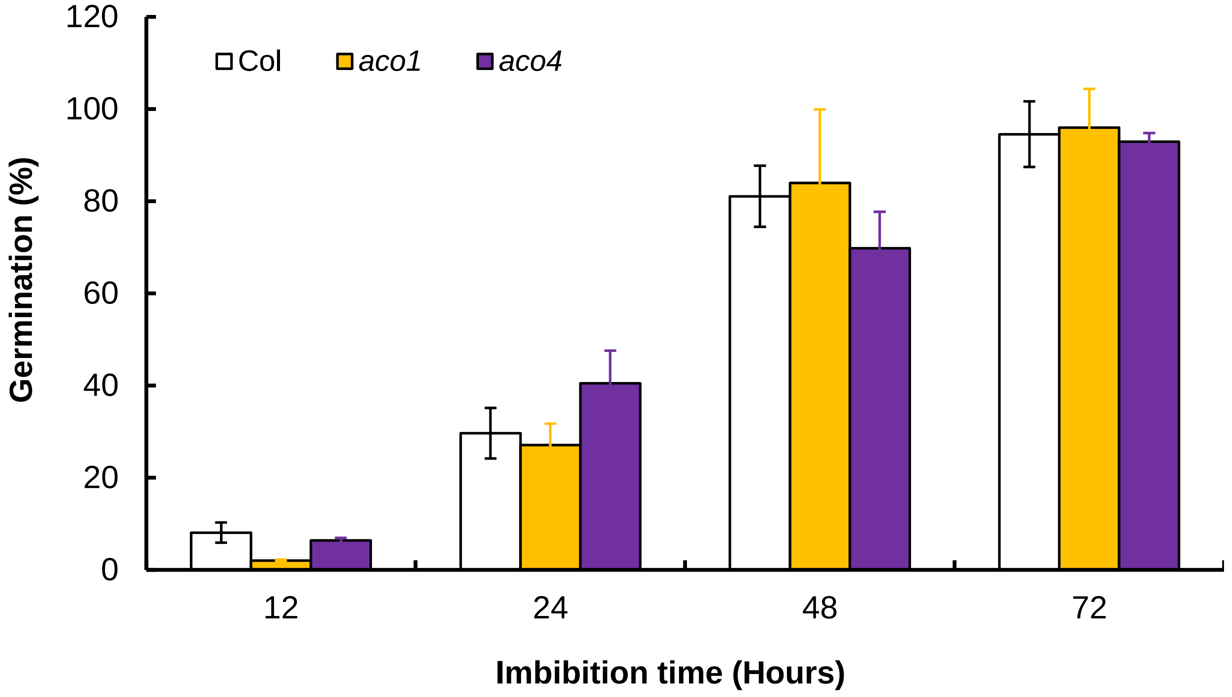


**Supplementary Figure 12. Germination phenotypes of *aco1* and *aco4* mutants.**
Germination during imbibition of after-ripened seeds from *aco1* and *aco4* mutants and the corresponding wild-type Col. The bars indicate standard deviation of eight biological replicates.

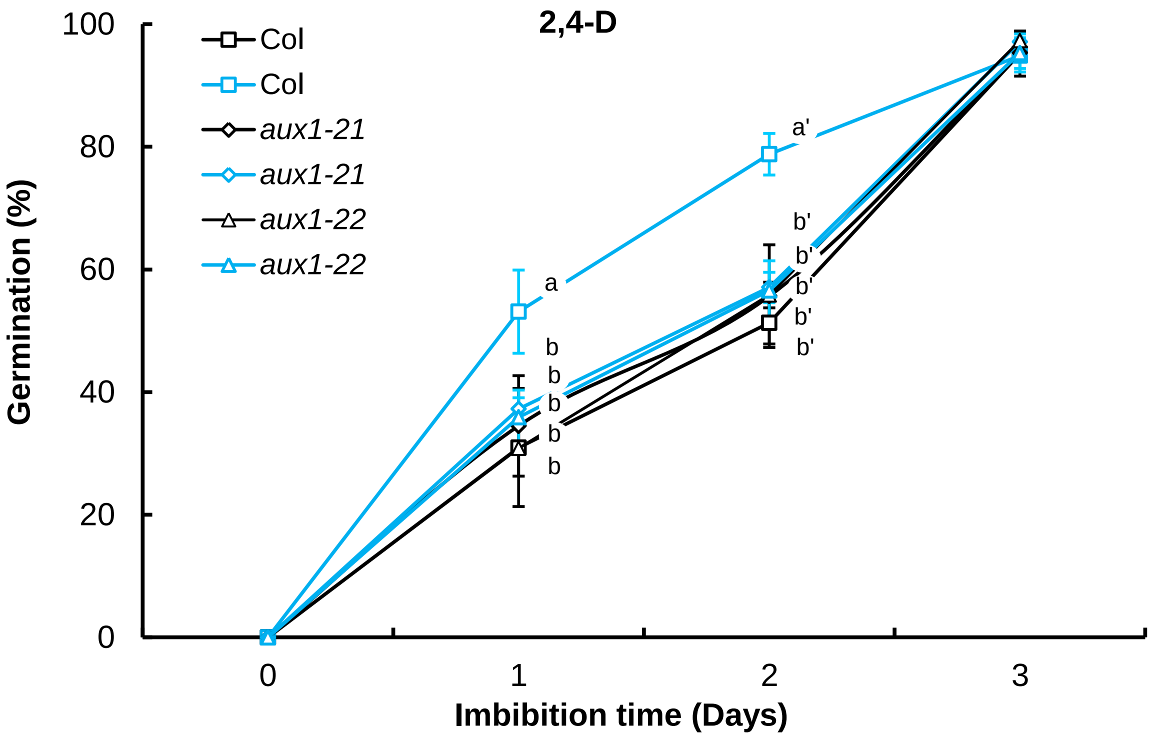


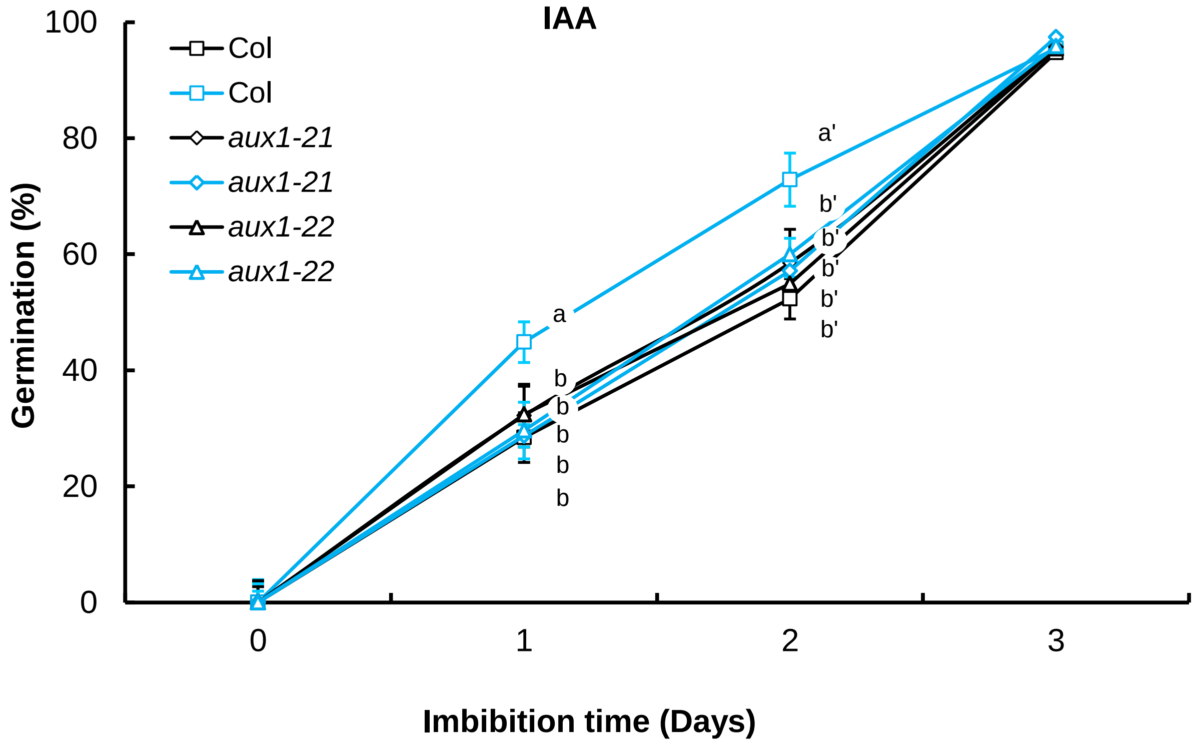


**Supplementary Figure 13. Seed germination phenotypes of *aux1* in response to low doses of auxin analogues.**
Exogenous application of 2,4-D or IAA at a low concentration (0.05 nM) promoted seed germination of Col significantly, but not *aux1* mutants. The seeds had been stored for 4 months at room temperature and completed their dormancy release. The black and blue lines indicate germination percentage changes in response to the mock treatments and 2,4-D (IAA) treatments respectively. Different letters at the same timepoint indicate a significant difference determined by Tukey's HSD test ($p$<0.05).

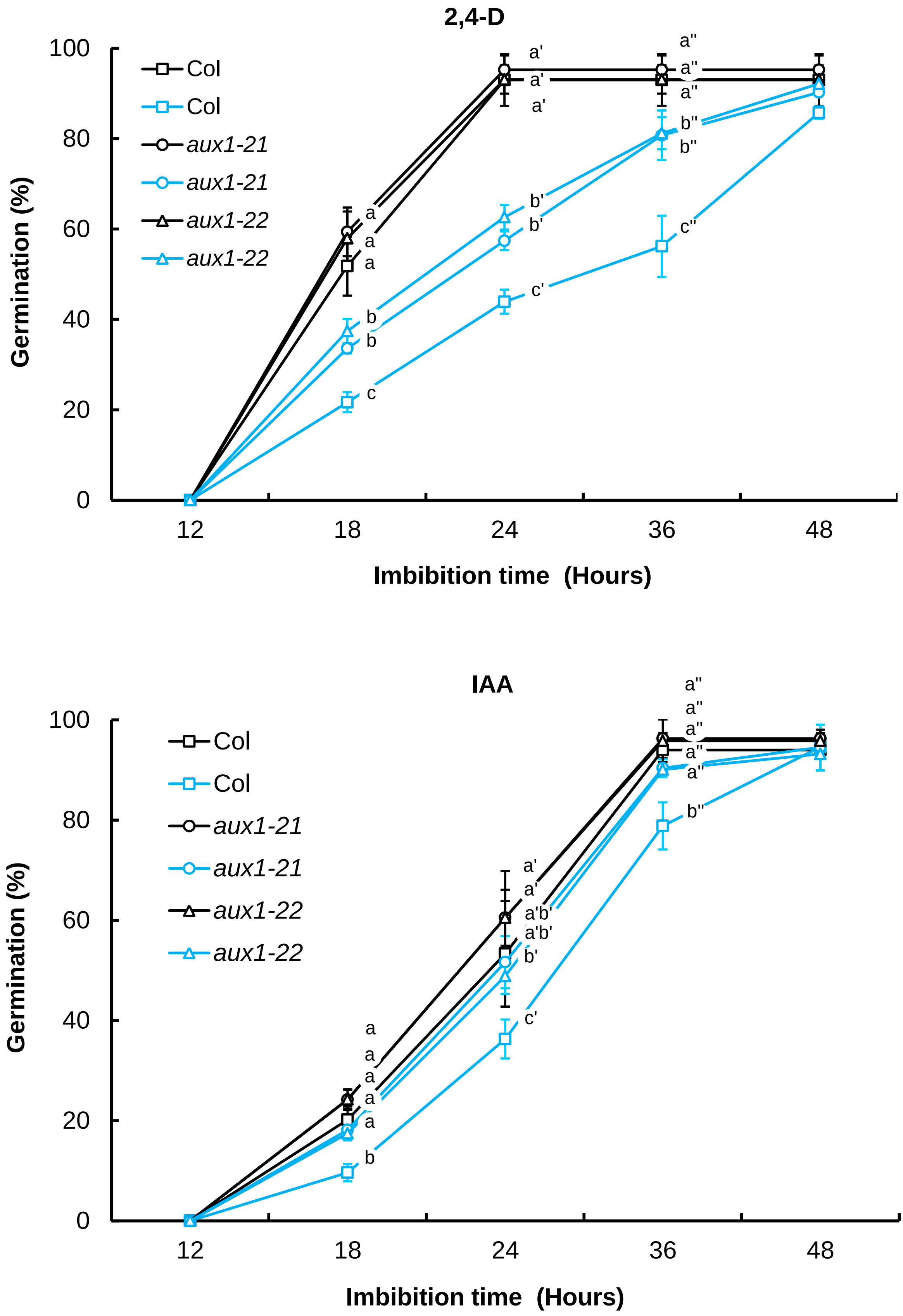


**Supplementary Figure 14. Seed germination phenotypes of *aux1* in response to high doses of auxin analogues.**
Exogenous application of 2,4-D or IAA at a high concentration (20 μM) repressed seed germination of Col more significantly than that of *aux1* mutants. The seeds had been stratified at 4 $^{o}$C and completed their dormancy release. The black and blue lines indicate germination percentage changes in response to the mock treatments and 2,4-D (IAA) treatments respectively. Different letters at the same timepoint indicate a significant difference determined by Tukey's HSD test ($p$<0.05).

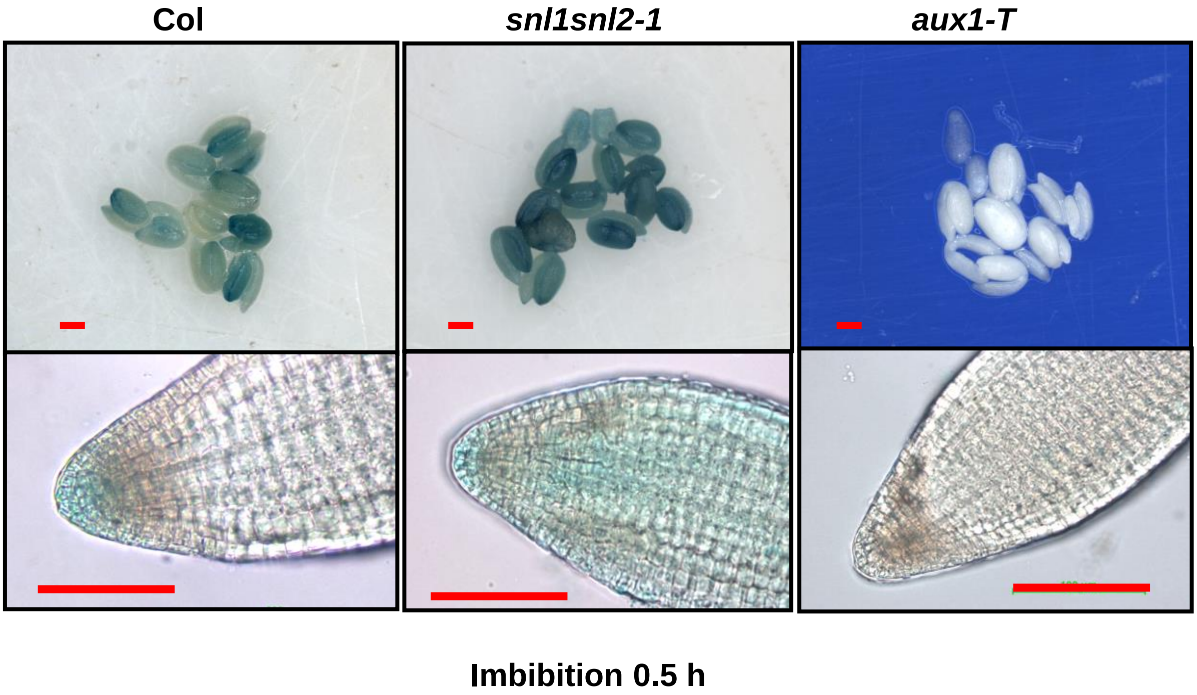


**Supplementary Figure 15. The loss of function *aux1-T* mutant shows reduced DR5::GUS signals.** The images show GUS stained embryos and radicles from *DR5::GUS/*Col, *DR5::GUS/snl1snl2*, and *DR5::GUS/aux1-T* seeds imbibed for 0.5 h. The experiment was performed twice with independent lines and similar results were obtained. Bar = 200 μm (upper panel), Bar = 100 μm (lower panel).

Figure 5a

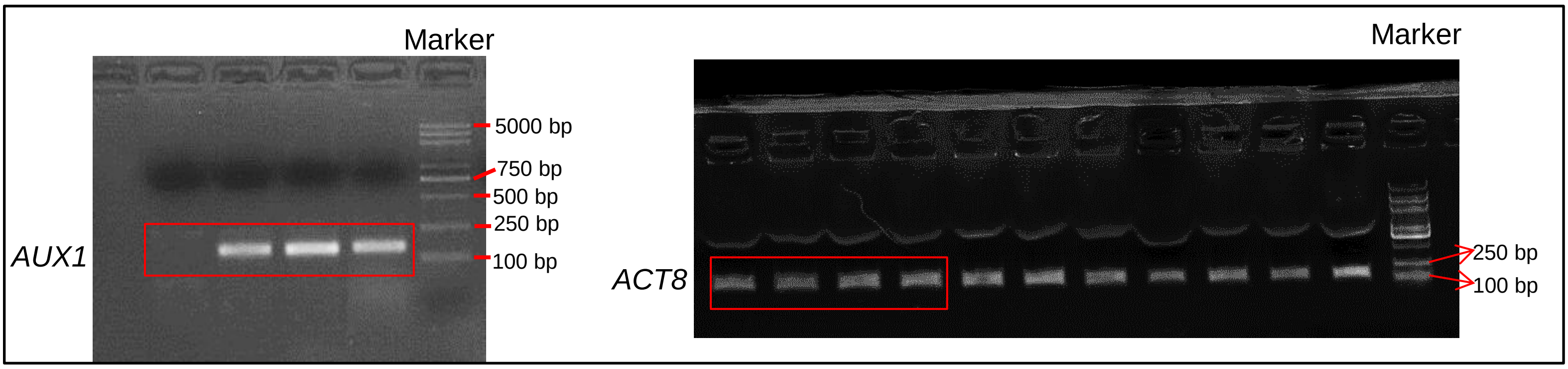


Figure 6b

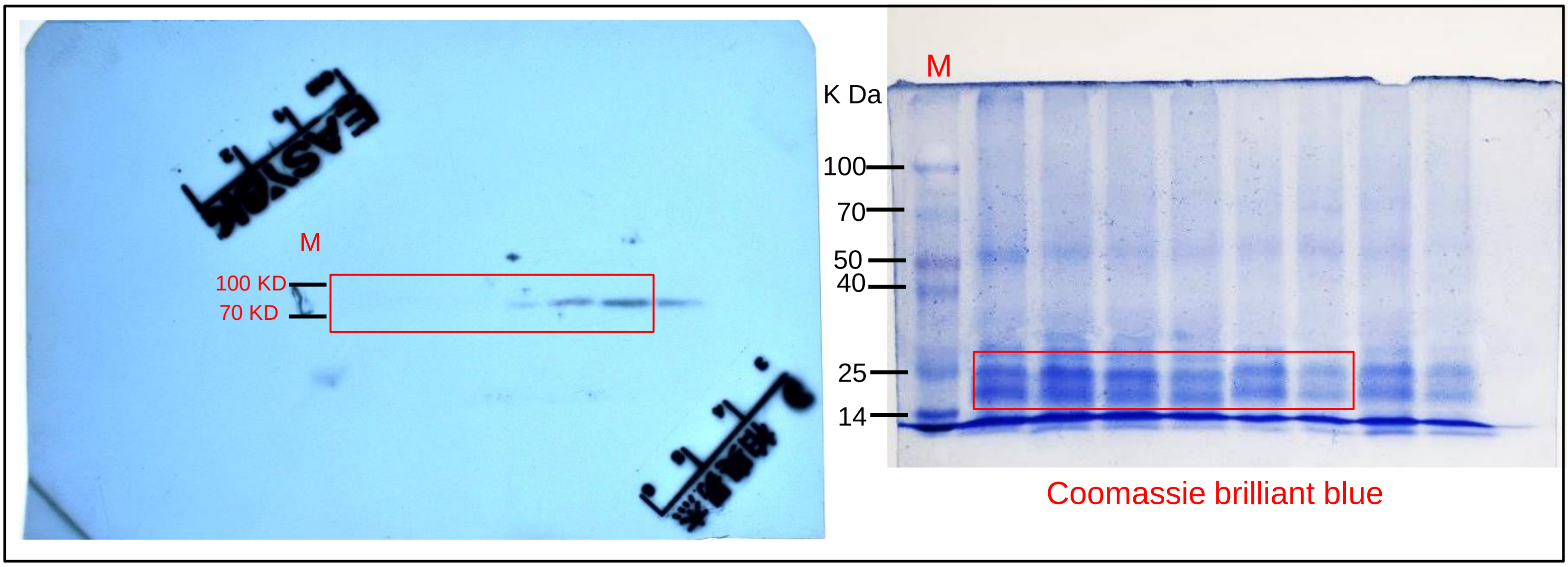


**Supplementary Figure 16. Uncropped DNA gels and immunoblots used in main figures.**
Red boxes indicate the cropped regions. Marker, DNA molecular weight; M, Pre-stained protein marker.

## Supplementary Tables

| Gene Function Annotation | Gene Name | Locus identifier | Expression level | Fold _Change (log2) | FDR (False discovery rate) |
|---|---|---|---|---|---|
| **Genes involved in the auxin metabolism** | | | | | |
| *CYTOCHROME P450, FAMILY 79, SUBFAMILY B, POLYPEPTIDE 2* | *CYP79B2* | AT4G39950 | Up | 3.8 | 1.84E-06 |
| *CYTOCHROME P450, FAMILY 79, SUBFAMILY B, POLYPEPTIDE 2* | *CYP79B3* | AT2G22330 | Up | 2.7 | 1.86E-05 |
| *YUCCA3* | *YUC3* | AT1G04610 | Up | 5 | 0.01281 |
| *TRYPTOPHAN BIOSYNTHESIS 4* | *TRP4* | AT1G25220 | Up | 1.5 | 3.76E-09 |
| *TRYPTOPHAN BIOSYNTHESIS 5* | *TRP5* | AT5G05730 | Up | 1.4 | 1.03E-09 |
| **Genes involved in auxin transport** | | | | | |
| *ENHANCER OF PINOID* | *ENP* | AT4G31820 | Up | 2.5 | 9.39E-05 |
| *ARABIDOPSIS THALIANA ATP-BINDING CASSETTE B19* | *ATABCB19* | AT3G28860 | Up | 1.9 | 6.97E-11 |
| *AUXIN RESISTANT 1* | *AUX1* | AT2G38120 | Up | 2.4 | 1.64E-08 |
| *ARABIDOPSIS THALIANA AMINOPEPTIDASE P1* | *ATAPP1* | AT4G36760 | Up | 1.0 | 1.82E-11 |
| *ARABIDOPSIS PIN-FORMED 3* | *ATPIN3* | AT1G70940 | Up | 1.6 | 8.42E-05 |
| *ARABIDOPSIS THALIANA PIN-FORMED 2* | *ATPIN2* | AT5G57090 | Up | 2.3 | 1.27E-07 |
| *ARABIDOPSIS THALIANA PIN-FORMED 1* | *ATPIN1* | AT1G73590 | Up | 1.2 | 4.59E-08 |
| **Genes involved in auxin homeostasis** | | | | | |
| *GH3.5* | *GH3.5* | AT4G27260 | Up | 1.8 | 1.19E-09 |
| *ALTERED TRYPTOPHAN REGULATION 4* | *ATR4* | AT4G31500 | Up | 3.7 | 2.07E-12 |
| *DWARF IN LIGHT 1* | *DFL1* | AT5G54510 | Up | 4.9 | 0.000393 |

(to be continued)

| **Genes involved in auxin signalling** | | | | | |
|---|---|---|---|---|---|
| *INDOLEACETIC ACID-INDUCED PROTEIN 1* | *IAA1* | AT4G14560 | UP | 5.3 | 0.0159979 |
| *INDOLEACETIC ACID-INDUCED PROTEIN 7* | *IAA7* | AT3G23050 | UP | 6.2 | 0.0446 |
| *INDOLEACETIC ACID-INDUCED PROTEIN 16* | *IAA16* | AT3G04730 | UP | 2.4 | 2.07E-09 |
| *INDOLEACETIC ACID-INDUCED PROTEIN 19* | *IAA19* | AT3G15540 | UP | 4.8 | 0.0424124 |
| *AUXIN RESPONSE FACTOR 5* | *ARF5* | AT1G19850 | Up | 1.6 | 3.28E-06 |

**Supplementary Table 1.** Auxin related genes with altered expression in *snl1snl2* seeds, identified by RNA-Seq analysis. Fold-change ≥ 2 (log2 Ratio ≥ 1) and false discovery rate (FDR) ≤ 0.05 were used as thresholds for significant gene expression differences.

| Primer name | DNA sequence |
|---|---|
| 900B12-LP | 5’-AATAGCACTGCCGGATGTATG-3’ |
| 900B12-RP | 5’-GAACACAAGCCTGAACTCGA-3’ |
| AUX1-F | 5’-CACCATGTCGGAAGGAGTAGAAGC-3’ |
| AUX1-R | 5’-TCAAAGACGGTGGTGTAAAGCG-3’ |
| AUX1-PF | 5’-CCTCCTCTGCACATTTCTGG-3’ |
| AUX1-PR | 5’-TCCGTTTGAGCTGTCACTTG-3’ |
| AUX1-QF(2) | 5’-TTCAGCTGCGCATCTAACCAA-3’ |
| AUX1-QR(2) | 5’-TCTGTATTCGACGTAGAGAACAG-3’ |
| AUX1-CF(3) | 5’-TACATATTTGGCGCGTGTTG-3’ |
| AUX1-CR(3) | 5’-ACCAAGCGGTGTAAGTGGTC-3’ |
| AUX1-CF(4) | 5’-TTATGGGTGCTGCTGTATCG-3’ |
| AUX1-CR(4) | 5’-TGACATTTCGCAAAAGCGTA-3’ |
| AUX1-CF(5) | 5’-CCACTCCAACGCTTTCTCTC-3’ |
| AUX1-CR(5) | 5’-ACTGCACGAACAAGAACACG-3’ |
| AUX1-CF(6) | 5’-CCTCCGCTCGTCAGgtataa-3’ |
| AUX1-CR(6) | 5’-TTTTTGCCCTCCAATTTCAC-3’ |
| AUX1-CF(7) | 5’-GCGATGTACGTGTTGAATGC-3’ |
| AUX1-CR(7) | 5’-GGCTGCAGCTGGTTTACATT-3’ |
| PIN2-PF | 5’-CAAAATGCCGAGGAAGAAAA-3’ |
| PIN2-PR | 5’-CGGCGAGAGAGAGAAGAAGA-3’ |
| PIN2-QF(2) | 5’-CCTCGCCGCACTCTTTCTTTGG-3’ |
| PIN2-QR(2) | 5’-CCGTACATCGCCCTAAGCAATGG-3’ |
| PIN2-CF(3) | 5’-CGAAGAAAGCAGGAAGAGGA-3’ |
| PIN2-CR(3) | 5’-ACCGGTGACTGATGGAAAAT-3’ |
| PIN3-QF | 5’-GAGATCCGTATGTTAGTCCC-3’ |
| PIN3-QR | 5’-GGCGTCTTTTGGTCTCTCTG-3’ |
| ATR4-QF | 5’-TTCATGAACGAGCACAAAGG-3’ |
| ATR4-QR | 5’-CATTGCAATCCCAAGATGC-3’ |
| IAA19-QF | 5’-GTGGGGTTAGGGTATGTGAA-3’ |
| IAA19-QR | 5’-ACCATCTTTCAAGGCCACAC-3’ |
| CYP79B2-PF | 5’-GGCAGGTCACCAACAAAACT-3’ |
| CYP79B2-PR | 5’-AGGGAGGGTCCATCTTGAGT-3’ |
| CYP79B2-CF(2) | 5’-TTATCTGCCGATGCTCACTG-3’ |
| CYP79B2-CR(2) | 5’-TAAGCAATGGGTTGCCTTGT-3’ |
| CYP79B2-QF(3) | 5’-CTCGCGAGACTTCTTCAAGG-3’ |
| CYP79B2-QR(3) | 5’-CCATAACCAACGGTTTAGCC-3’ |
| CYCD1;1-QF | 5’-CAAGATCGATCCTTCGGGTA-3’ |
| CYCD1;1-QR | 5’-CACCAAGTCTCAGGGCTCTC-3’ |
| CYCD4;1-QF | 5’-GGCAGCCAAAATTGAAGAAA-3’ |
| CYCD4;1-QR | 5’-ACAGCAGCAGCAACTTCAGA-3’ |

(to be continued)

| | |
|---|---|
| YUC3-QF | 5’-CCGAAAATTGGACCGTTAGA-3’ |
| YUC3-QR | 5’-ATACAATCCCGCCTCTCCTT-3’ |
| CYP79B3-QF | 5’-ACGTACGGCGAGGATAATTC-3’ |
| CYP79B3-QR | 5’-CAAAAGGACCAAAACCGAAC-3’ |
| ARF5-QF | 5’-GAAGCTGTTGAAAGACCAGT-3’ |
| ARF5-QR | 5’-CTGCTCGCTATGACCTTGTG-3’ |
| SNL1-QF2 | 5’-CGTCCTCTTGGTTCTTCTCG-3’ |
| SNL1-QR2 | 5’-TACGACAACGCATCATTGGT-3’ |
| SNL2-QF2 | 5’-CATAGGCAAATTCCGTCCTC-3’ |
| SNL2-QR2 | 5’-CTCGAGTAAGAGCGGAATCG-3’ |
| 12S-F | 5’-GGCGCGCCAACTTAAGAGCTTATGATG-3’ |
| 12S-R | 5’-GAAGGCCTTTCTTTTTGTTTGTTGTGAG-3’ |
| ACTIN8-QF | 5’-CTCAGGTATTGCAGACCGTATGAG-3’ |
| ACTIN8-QR | 5’-CTGGACCTGCTTCATCATACTCTG-3’ |

QF and QR: Primer for quantitative PCR

PF and PR: primer for ChIP qRT-PCR, amplifying fragment in promoter (region 1)

CF and CR: primer for ChIP qRT-PCR, amplifying fragment in encoding region

(2): primer for ChIP, amplified fragment in encoding region 2

(3): primer for Chip, amplified fragment in encoding region 3

(4): primer for ChIP, amplified fragment in encoding region 4

(5): primer for ChIP, amplified fragment in encoding region 5

(6): primer for ChIP, amplified fragment in encoding region 6

(7): primer for ChIP, amplified fragment in encoding region 7

**Supplementary Table 2.** Primers for mutant genotyping, plasmid construction, qRT-PCR and ChIP assay.
